# Supplementary material for: Understanding inequalities in COVID-19 outcomes following hospital admission for people with intellectual disability compared to the general population: a matched cohort study in the UK
Source: BMJ Open. 2021 Oct 3;11(10):e052482. doi: 10.1136/bmjopen-2021-052482 (PMC8491000; doi:10.1136/bmjopen-2021-052482)

Appendix Table 1. Complications related to COVID-19 in hospitalised patients with and without an ID diagnosis

	Controls		ID group		p value of comparison
	n (%)	N	n (%)	N	
Viral pneumonia	595 (43.3)	1375	222 (47.4)	468	0.119
Bacterial pneumonia	153 (11.2)	1364	63 (13.6)	463	0.182
Acute respiratory syndrome	160 (11.6)	1382	44 (9.3)	471	0.201
Other lung complications[1]	97 (6.9)	1408	28 (5.9)	476	0.523
Meningitis / Encephalitis	6 (0.4)	1396	1 (0.2)	474	0.687
Seizures	28 (2.0)	1401	24 (5.1)	474	**0.001**
Other neurological complications[2]	31 (2.2)	1401	7 (1.5)	472	0.450
Cardiac arrest	31 (2.2)	1397	9 (1.9)	473	0.854
Other cardiac complications[3]	132 (9.4)	1409	34 (7.2)	473	0.160
Bacteraemia	40 (2.9)	1391	10 (2.1)	469	0.509
Gastrointestinal hemorrhage or coagulation disorder	44 (3.1)	1402	16 (3.4)	473	0.764
Pancreatitis	10 (0.7)	1395	1 (0.2)	473	0.309
Rhabdomyolysis / Myositis	5 (0.4)	1395	2 (0.4)	473	1.000
Anaemia	164 (11.7)	1404	46 (9.7)	473	0.273
Acute renal injury and/or acute renal failure	192 (13.7)	1402	57 (12.0)	475	0.389
Liver dysfunction	60 (4.3)	1396	16 (3.4)	472	0.422
Hypoglycaemia or hyperglycaemia	88 (6.3)	1386	30 (6.3)	473	1.000

[1]Combined cryptogenic organizing pneumonia (COP), pneumothorax, pleural effusion and bronchiolitis, [2]Combined Stroke / Cerebrovascular accident and other neurological complication, [3]Combined congestive heart failure, endocarditis / myocarditis pericarditis, myocarditis / pericarditis, cardiomyopathy, cardiac arrhythmia, cardiac ischemia. The number of patients in the ID group developing Covid-19 related complications while in hospital were compared to controls using Fisher's exact test.

Appendix Figure 1. Mortality rates of hospitalised COVID-19 patients with and without an intellectual disability diagnosis

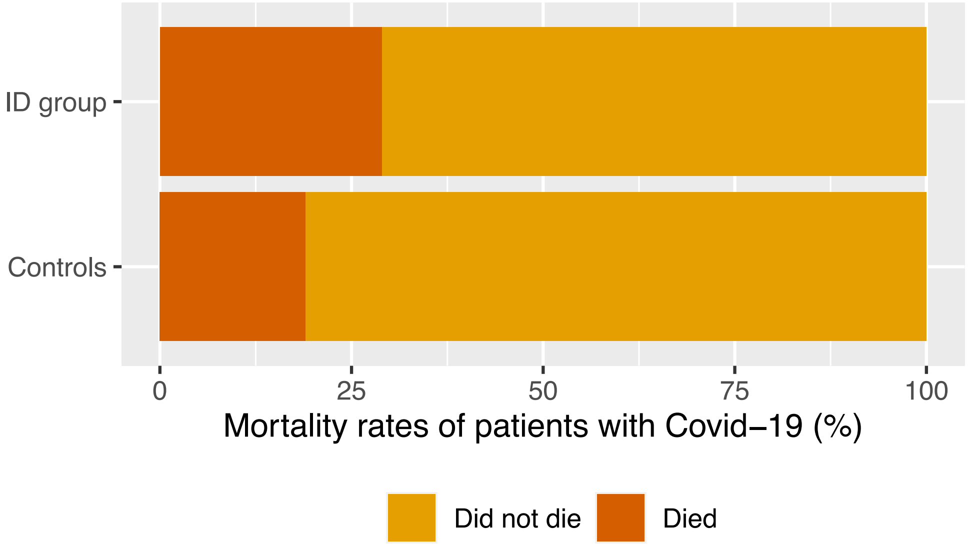

Appendix Table 2. Factors associated with mortality in hospitalised COVID-19 patients

	Risk ratio	95% CI	p value
Age group			
20-29 years old	3.39	0.40 - 44.51	0.300
30-39 years old	6.95	1.29 - 61.92	*0.064*
40-49 years old	10.17	2.06 - 72.36	**0.023**
50-59 years old	22.22	5.44 - 90.88	**0.001**
60-69 years old	25.94	6.57 - 93.93	**0.0006**
70-79 years old	37.26	10.31 - 100.09	**0.0001**
80+ years old	60.18	20.83 - 106.13	**<0.0001**
Male sex	1.18	0.918 - 1.50	0.191
Chronic cardiac disease	1.35	1.02 - 1.76	**0.038**
Chronic pulmonary disease	1.66	1.22 - 2.16	**0.002**
Chronic kidney disease	1.58	1.13 - 2.12	**0.009**
Liver disease	1.69	0.97 - 2.54	*0.055*
Obesity	1.21	0.97 - 1.62	0.242
Chronic neurological disorder	1.64	1.23 - 2.11	**0.001**
Dementia	1.11	0.71 - 1.64	0.632
Malignant neoplasm	1.32	0.84 - 1.92	0.209
Shortness of breath	0.96	0.70 - 1.29	0.785
Respiratory rate	1.03	1.02 - 1.05	**0.0003**
No oxygen therapy	0.72	0.55 - 0.91	**0.005**
Admission to ICU	0.92	0.51 - 1.51	0.758
Intubation	3.11	2.22 - 3.98	**<0.0001**
Non-invasive respiratory support	1.44	1.02 - 1.95	**0.039**
DS diagnosis	1.92	1.19 - 2.76	**0.009**
ID diagnosis	1.56	1.17 - 2.02	**0.003**

ICU, Intensive Care Unit; DS, Down syndrome, ID; Intellectual disability. Tracheostomy was not included in the model due to a large proportion of missing data.

Appendix Table 3. Factors associated with mortality in hospitalised COVID-19 patients with an intellectual disability diagnosis

	Risk ratio	95% CI	p value
Age group			
20-29 years old	1.12	0.13 - 4.99	0.903
30-39 years old	2.41	0.61 - 6.58	0.213
40-49 years old	2.85	0.80 – 7.00	0.120
50-59 years old	4.28	1.62 - 8.04	**0.012**
60-69 years old	6.43	3.21 - 9.07	**0.0002**
70-79 years old	4.04	1.40 - 7.93	**0.022**
80+ years old	7.33	3.74 - 9.44	**0.0001**
Male sex	1.24	0.84 - 1.71	0.267
DS diagnosis	1.41	0.86 - 2.02	0.152
Chronic cardiac disease	1.50	0.94 - 2.12	*0.085*
Chronic pulmonary disease	1.08	0.55 - 1.78	0.789
Chronic kidney disease	1.50	0.85 - 2.22	0.142
Liver disease	1.07	0.33 - 2.20	0.894
Obesity	0.93	0.48 - 1.52	0.803
Chronic neurological disorder	1.39	0.94 - 1.90	*0.091*
Dementia	1.25	0.66 - 2.01	0.454
Malignant neoplasm	0.81	0.28 - 1.69	0.633
Shortness of breath	0.99	0.62 - 1.47	0.960
Respiratory rate	1.02	1.00 - 1.05	**0.036**
No oxygen therapy	0.49	0.31 - 0.73	**0.0002**
Access to any intervention	1.54	0.99 - 2.15	*0.054*

Appendix Table 4. Associations between complications due to COVID-19 and mortality in patients with an ID diagnosis

	Risk ratio	95% CI	*p value*
Age group			
20-29 years old	2.39	0.22 - 22.71	0.478
30-39 years old	4.78	0.79 - 27.72	0.141
40-49 years old	7.00	1.39 - 30.29	*0.053*
50-59 years old	7.92	1.77 - 30.94	**0.032**
60-69 years old	14.97	4.16 -34.30	**0.003**
70-79 years old	7.37	1.52 - 30.59	**0.045**
80+ years old	18.11	5.11 -35.12	**0.001**
Male sex	1.11	0.77 - 1.54	0.550
Viral pneumonia	2.74	1.97 - 3.60	**<0.0001**
Bacterial pneumonia	1.60	0.98 - 2.29	*0.054*
Acute respiratory syndrome	2.07	1.28 - 2.88	**0.006**
Other lung complications[1]	1.76	0.93 -2.	*0.077*
Seizures	0.39	0.06 - 1.16	0.146
Other neurological complications[2]	0.87	0.15 - 2.50	0.844
Other cardiac complications[3]	0.64	0.24 -1.34	0.278
Bacteraemia	1.57	0.42 - 3.02	0.432
Gastrointestinal hemorrhage or coagulation disorder	0.51	0.12 -1.48	0.267
Anaemia	0.51	0.20 - 1.08	*0.096*
Acute renal injury / Acute renal failure	1.17	0.62 - 1.92	0.594
Liver dysfunction	0.90	0.21 - 2.11	0.851
Hypoglycaemia or hyperglycaemia	0.53	0.17 -1.25	0.183

[1]Combined cryptogenic organizing pneumonia (COP), pneumothorax, pleural effusion and bronchiolitis, [2]Combined Stroke / Cerebrovascular accident and other neurological complication, [3]Combined congestive heart failure, endocarditis / myocarditis pericarditis, myocarditis / pericarditis, cardiomyopathy, cardiac arrhythmia, cardiac ischemia. Meningitis, pancreatitis and rhabdomyolysis where removed from the model because they were recorded in less than 1% of ID patients. Ethnicity and cardiac arrest were also removed because they were not good predictors in the model.

Appendix Table 5. Associations between complications due to COVID-19 and mortality in patients without an ID diagnosis

	Risk ratio	95% CI	*p value*
Age group			
20-29 years old	1.07	0.042 - 22.07	0.961
30-39 years old	2.04	0.30 - 29.80	0.522
40-49 years old	4.12	0.75 - 45.82	0.179
50-59 years old	10.34	2.34 - 69.97	**0.018**
60-69 years old	13.12	3.05 - 76.02	**0.008**
70-79 years old	26.96	7.05 -91.13	**0.0005**
80+ years old	36.49	10.18 - 95.93	**0.0001**
Male sex	1.02	0.75 - 1.35	0.913
Viral pneumonia	1.56	1.16 - 2.07	**0.003**
Bacterial pneumonia	1.01	0.63 - 1.52	0.970
Acute respiratory syndrome	1.91	1.32 - 2.62	**0.0008**
Other lung complications[1]	1.11	0.64 - 1.79	0.683
Seizures	0.97	0.32 - 2.23	0.958
Other neurological complications[2]	0.93	0.33 - 2.06	0.881
Cardiac arrest	5.38	3.94 - 6.15	**<0.0001**
Other cardiac complications[3]	1.82	1.22 - 2.57	**0.004**
Bacteraemia	0.82	0.32 - 1.75	0.646
Gastrointestinal hemorrhage or coagulation disorder	2.78	1.60 - 4.09	**0.0009**
Anaemia	1.23	0.80 - 1.80	0.316
Acute renal injury / Acute renal failure	1.99	1.41 - 2.69	**0.0002**
Liver dysfunction	0.50	0.21 - 1.03	*0.072*
Hypoglycaemia or hyperglycaemia	1.15	0.64 - 1.90	0.620

[1]Combined cryptogenic organizing pneumonia (COP), pneumothorax, pleural effusion and bronchiolitis, [2]Combined Stroke / Cerebrovascular accident and other neurological complication, [3]Combined congestive heart failure, endocarditis / myocarditis pericarditis, myocarditis / pericarditis, cardiomyopathy, cardiac arrhythmia, cardiac ischemia. Meningitis, pancreatitis and rhabdomyolysis where removed from the model because they were recorded in less than 1% of control patients. Ethnicity was removed because it was not a good predictor in the model.

Appendix Table 6. Kaplan-Meier estimates of survival probability of hospitalised COVID-19 patients with and without an intellectual disability diagnosis

Time in hospital (days)	Survival probability (% and 95% CI)	
	Controls (n = 1484)	ID group (n = 472)
0	99.6 (99.3 - 99.9)	98.7 (97.7 - 99.7)
5	93.5 (92.1 - 95.0)	83.4 (79.9 - 87.0)
10	83.0 (80.5 - 85.6)	70.9 (66.5 - 75.7)
15	73.6 (70.3 - 77.0)	65.6 (60.7 - 70.8)
20	67.3 (63.5 - 71.3)	60.7 (55.4 - 66.6)

Appendix Figure 2. Violin plot of the distribution of length of stay in COVID-19 patients with and without an intellectual disability diagnosis who were discharged alive

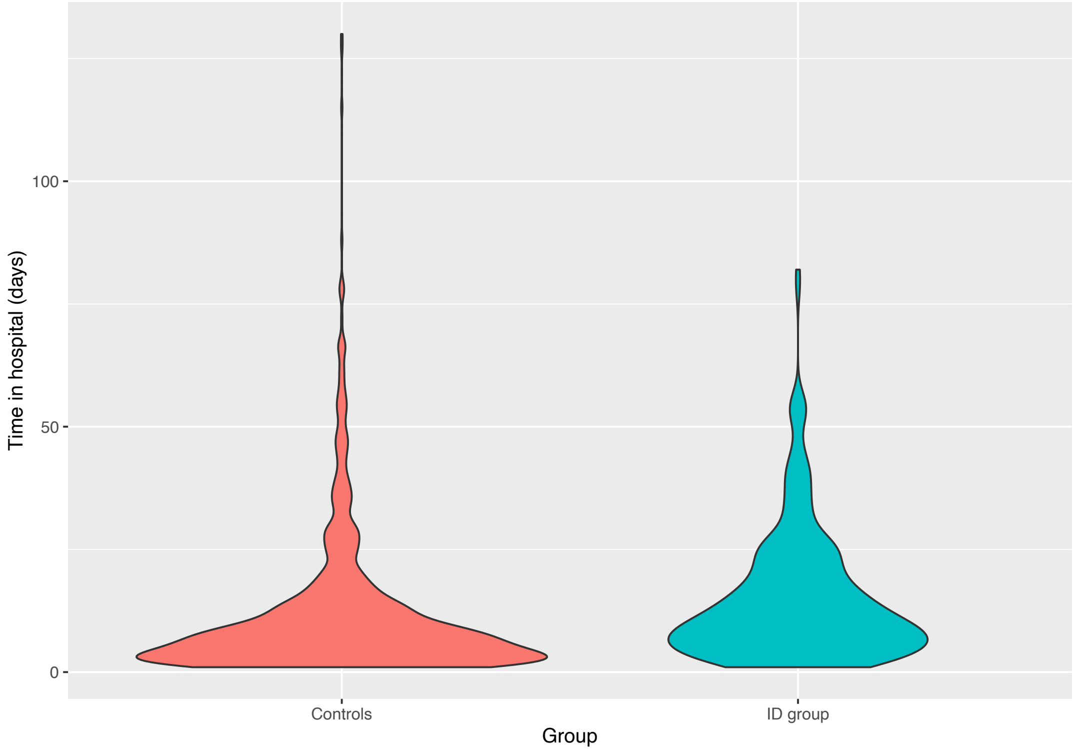

Supplement: Supplementary data [file bmjopen-2021-052482supp001.pdf]
