# Supplementary material for: Understanding inequalities in COVID-19 outcomes following hospital admission for people with intellectual disability compared to the general population: a matched cohort study in the UK
Source: BMJ Open. 2021 Oct 3;11(10):e052482. doi: 10.1136/bmjopen-2021-052482 (PMC8491000; doi:10.1136/bmjopen-2021-052482)

made with photosymbols®

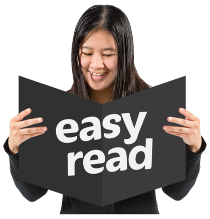

symptoms of Covid-19 and hospital treatments in people with and without a learning disability

easy read summary

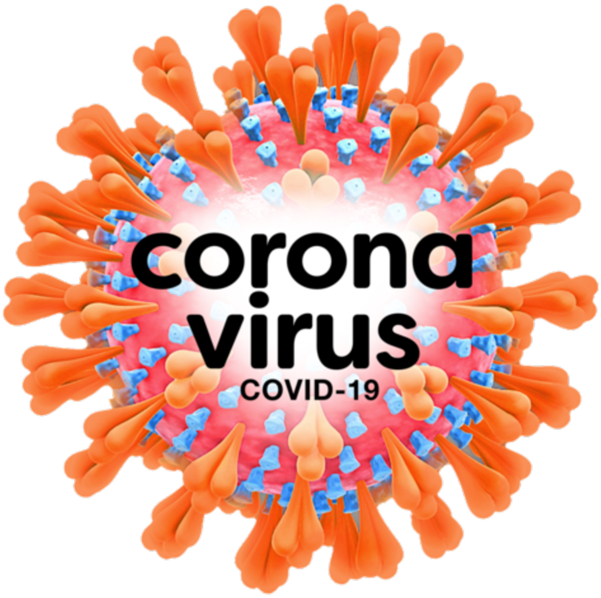

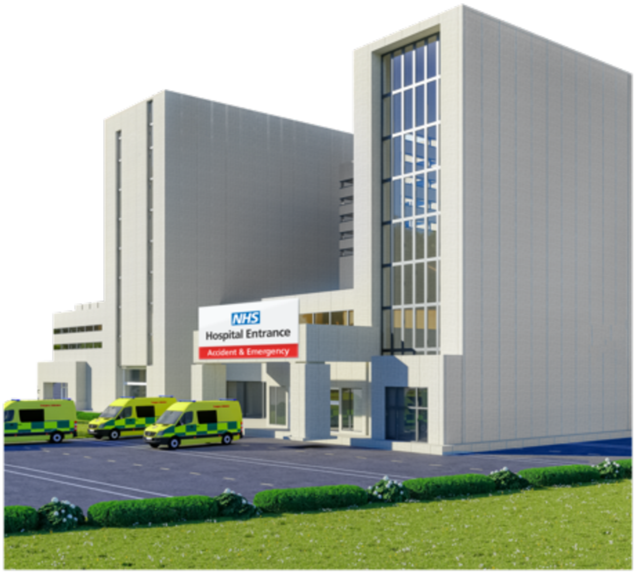

Based on findings from:
Baksh RA, Pape SE, Smith J, et al. Understanding inequalities in COVID-19 outcomes following hospital admission for people with intellectual disability compared to the general population: a matched cohort study in the UK, BMJ Open 2021;11:e052482. doi: 10.1136/bmjopen-2021-052482

background

covid-19 is a virus

a virus can make people feel unwell

a virus can also cause people to die

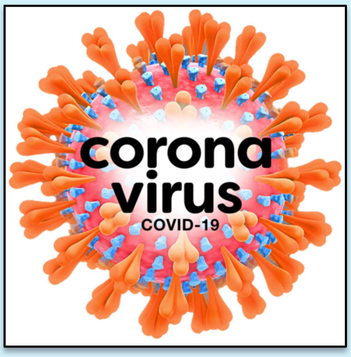

in 2020 lots of people became unwell because of covid-19

people had bad coughs, felt hot, and found it hard to breathe

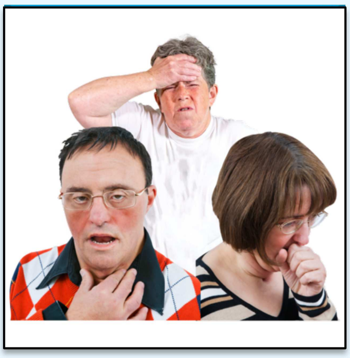

some people were very sick and needed to go to hospital

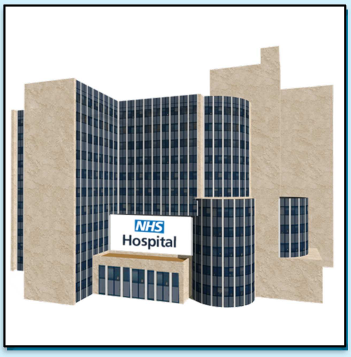

we wanted to find out what happened to people in the UK who went to hospital because of covid-19

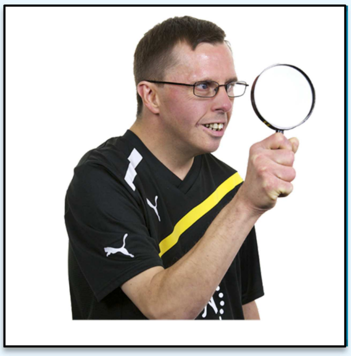

what we did

we used information from hospitals all over the UK

the information was from the start of the covid-19 pandemic

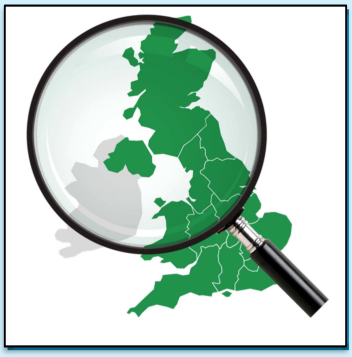

we looked at information about 506 people with a learning disability and 1518 people without a learning disability

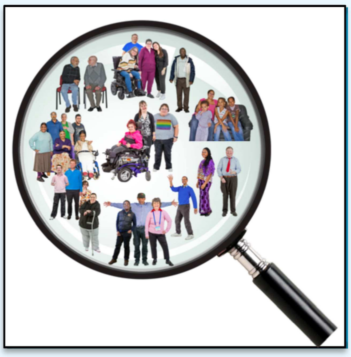

the people in the study with a learning disability were matched with people without a learning disability who were the same age, sex and ethnicity

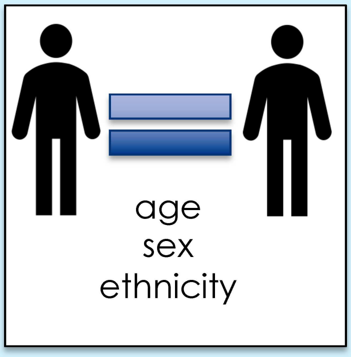

we looked to see whether there were differences between treatments for people with a learning disability and people without a learning disability

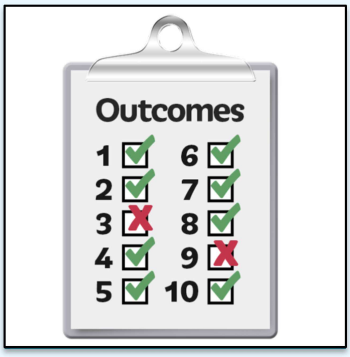

what we found out

people with a learning disability were less likely to have changes in smell or taste and were less likely to report pain when they had covid-19

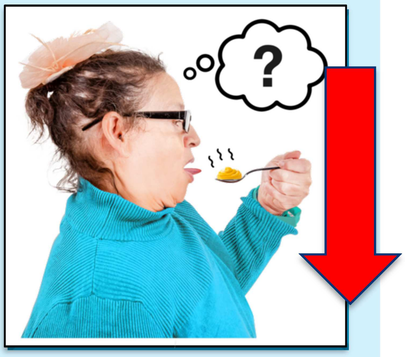

people with a learning disability were more likely to be confused, sleepy, or have fits when they had covid-19

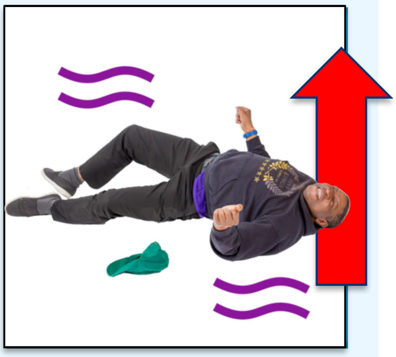

people with a learning disability were more unwell when they arrived at hospital

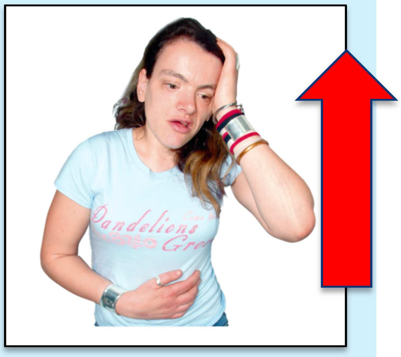

they had faster breathing and needed oxygen more often

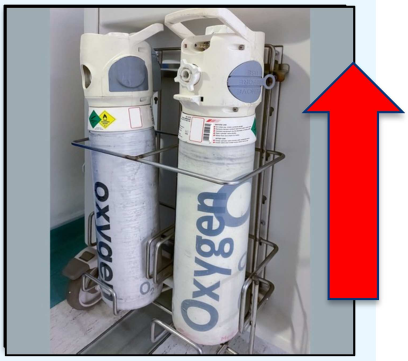

what we found out

fewer people with a learning disability were given ventilation – this is used to help people breathe

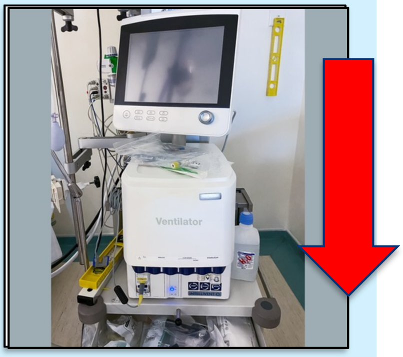

they were also admitted to intensive care units less often than people without a learning disability

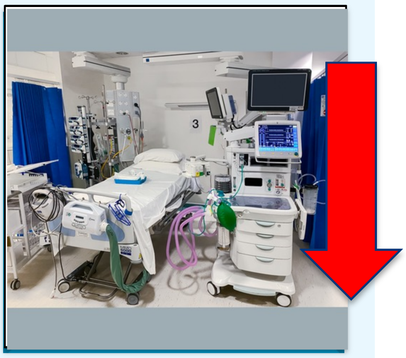

people with learning disabilities died more quickly and stayed in hospital for longer than people without a learning disability

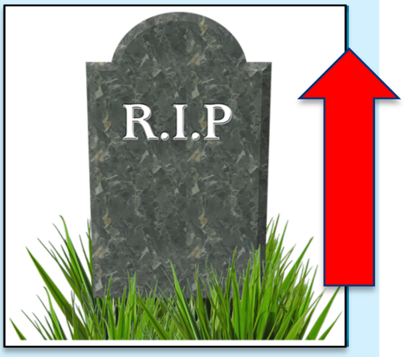

this is even though people with learning disabilities did **not** have more health problems than people without a learning disability

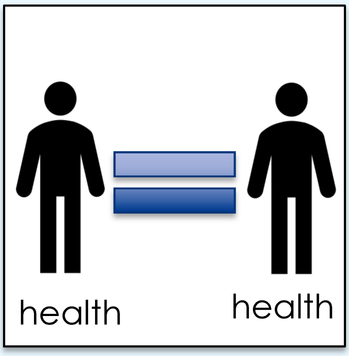

important messages

we found that people with a learning disability did worse than people without a learning disability with covid-19 in hospital

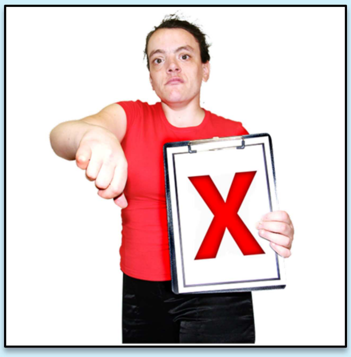

we are worried that this is because people with learning disabilities were not given the same treatments as other people

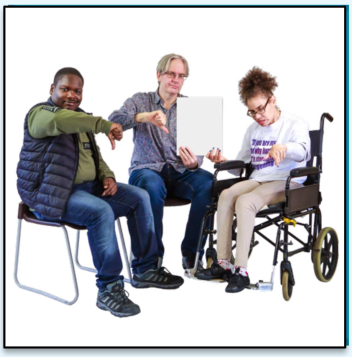

we recommend that the needs of people with learning disabilities are better supported so they can access the same treatment as everyone else

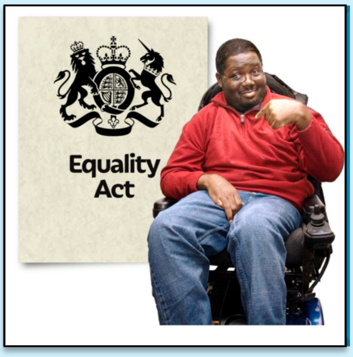

people with a learning disability should also be helped to have treatments in the community that will keep them from getting sick

this includes having access to the covid-19 vaccine

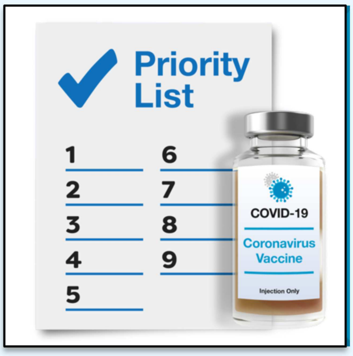

Supplement: Supplementary data [file bmjopen-2021-052482supp002.pdf]
