# Supplementary material for: Understanding inequalities in COVID-19 outcomes following hospital admission for people with intellectual disability compared to the general population: a matched cohort study in the UK
Source: BMJ Open. 2021 Oct 3;11(10):e052482. doi: 10.1136/bmjopen-2021-052482 (PMC8491000; doi:10.1136/bmjopen-2021-052482)
Supplement: Author's manuscript [file bmjopen-2021-052482.draft_revisions.pdf]

BMJ Open is committed to open peer review. As part of this commitment we make the peer review history of every article we publish publicly available.

When an article is published we post the peer reviewers' comments and the authors' responses online. We also post the versions of the paper that were used during peer review. These are the versions that the peer review comments apply to.

The versions of the paper that follow are the versions that were submitted during the peer review process. They are not the versions of record or the final published versions. They should not be cited or distributed as the published version of this manuscript.

BMJ Open is an open access journal and the full, final, typeset and author-corrected version of record of the manuscript is available on our site with no access controls, subscription charges or pay-per-view fees (http://bmjopen.bmj.com).

If you have any questions on BMJ Open's open peer review process please email info.bmjopen@bmj.com

BMJ Open

Understanding inequalities in COVID-19 outcomes following hospital admission for people with Intellectual disability compared to the general population

Journal:	*BMJ Open*
Manuscript ID	bmjopen-2021-052482
Article Type:	Original research
Date Submitted by the Author:	16-Apr-2021

Complete List of Authors:	Baksh, R. Asaad; Institute of Psychiatry, Psychology, and Neuroscience, Department of Forensic and Neurodevelopmental Sciences, King's College London,<br>Pape, Sarah ; King's College London; South London and Maudsley NHS Foundation Trust<br>Smith, James; South London and Maudsley NHS Foundation Trust<br>Strydom, André; Institute of Psychiatry Psychology and Neuroscience

Keywords:	INTENSIVE & CRITICAL CARE, Public health < INFECTIOUS DISEASES, PUBLIC HEALTH

SCHOLARONE™
Manuscripts

I, the Submitting Author has the right to grant and does grant on behalf of all authors of the Work (as defined in the below author licence), an exclusive licence and/or a non-exclusive licence for contributions from authors who are: i) UK Crown employees; ii) where BMJ has agreed a CC-BY licence shall apply, and/or iii) in accordance with the terms applicable for US Federal Government officers or employees acting as part of their official duties; on a worldwide, perpetual, irrevocable, royalty-free basis to BMJ Publishing Group Ltd ("BMJ") its licensees and where the relevant Journal is co-owned by BMJ to the co-owners of the Journal, to publish the Work in this journal and any other BMJ products and to exploit all rights, as set out in our licence.

The Submitting Author accepts and understands that any supply made under these terms is made by BMJ to the Submitting Author unless you are acting as an employee on behalf of your employer or a postgraduate student of an affiliated institution which is paying any applicable article publishing charge ("APC") for Open Access articles. Where the Submitting Author wishes to make the Work available on an Open Access basis (and intends to pay the relevant APC), the terms of reuse of such Open Access shall be governed by a Creative Commons licence – details of these licences and which Creative Commons licence will apply to this Work are set out in our licence referred to above.

Other than as permitted in any relevant BMJ Author's Self Archiving Policies, I confirm this Work has not been accepted for publication elsewhere, is not being considered for publication elsewhere and does not duplicate material already published. I confirm all authors consent to publication of this Work and authorise the granting of this licence.

For peer review only

Understanding inequalities in COVID-19 outcomes following hospital admission for people with Intellectual disability compared to the general population

R. Asaad Baksh,[1,3] Sarah E. Pape [1,2,3], James Smith,[2] & Andre Strydom [1,2,3]

[1.] *Institute of Psychiatry, Psychology, and Neuroscience, King's College London, London, UK.*

[2.] *South London and Maudsley NHS Foundation Trust, London, UK.*

[3.] *The LonDowns Consortium, London, UK.*

For peer review only

Corresponding author: Dr R. Asaad Baksh
Department of Forensic and Neurodevelopmental Sciences, Institute of Psychiatry, Psychology & Neuroscience, King's College London, London, SE5 8AF, UK
Email: asaad.baksh@kcl.ac.uk

Manuscript text: 3331 words (excluding, abstract, figures and tables)

ABSTRACT

OBJECTIVES: This study explores the hospital journey of patients with Intellectual disabilities (ID) compared to the general population after admission for COVID-19 during the first wave of the pandemic (when demand on inpatient resources was high) to identify disparities in treatment and outcomes.

DESIGN: Matched cohort study; an ID cohort of 506 patients were matched based on age, sex and ethnicity with a control group using a 1:3 ratio to compare outcomes from the International Severe Acute Respiratory and emerging Infections Consortium (ISARIC) World Health Organization (WHO) Clinical Characterisation Protocol UK (CCP-UK).

SETTING: Admissions for COVID-19 from U.K hospitals; data on symptoms, severity, access to interventions, complications, mortality and length of stay were extracted.

INTERVENTIONS: Non-invasive respiratory support, intubation, tracheostomy, ventilation and admission to intensive care units (ICU).

RESULTS: Subjective presenting symptoms of COVID-19 such as loss of taste/smell were less frequently reported in ID patients, whereas indicators of more severe disease such as altered consciousness and seizures were more common. ID patients were admitted with higher respiratory rates (Median = 22, range = 10-48) and were more likely to require oxygen therapy (35.1% vs 28.9%) compared to controls. Despite this, ID patients were 37% (13% - 57% 95% CI) less likely to receive non-invasive respiratory support, 40% (7% - 63% 95% CI) less likely to receive intubation and 50% (30% – 66% 95% CI) less likely to be admitted to the ICU while in hospital. They had a 56% (17% - 102% 95% CI) increased risk of dying from COVID-19 after they were hospitalised and were dying 1.44 times faster (1.13 – 1.84 95% CI) compared to controls.

CONCLUSIONS: There have been significant disparities in healthcare between people with ID and the general population during the COVID-19 pandemic, which may have contributed to excess mortality in this group.

For peer review only

ARTICLE SUMMARY

STRENGTHS AND LIMITATIONS OF THIS STUDY

- This is the first in-depth analysis of the hospital journey of patients with Intellectual disabilities compared to the general population after admission for COVID-19.
- We had a large sample size and well-matched controls with data on symptoms, treatment and outcomes related to COVID-19.
- Our results offer important insights into conditions faced by patients and health professionals during the first wave of the COVID-19 pandemic.
- Due to data being collected at the time of care there was some degree of missing or incomplete data.

For peer review only

INTRODUCTION

Intellectually disability (ID) affects around 1% of the population globally and results in varying degrees of functional impairments depending on the severity of ID [1]. Poorer health outcomes compared to the general population have been consistently reported for people with ID [2], with an increased incidence of comorbidities including dysphagia and respiratory diseases, with respiratory disease identified as a leading cause of death [3]. These health comorbidities are associated with poor outcomes following infections and other acute conditions[4 5], which may be exacerbated by barriers in accessing health and social care, associated with concerns about ongoing discrimination and bias [6].

To date there have been over 64 million cases of COVID-19 reported worldwide and 1.4 million deaths [7]. Several risk factors for increased mortality have been identified and reported [8], including increasing age [9], cardiovascular disease, chronic lung disease [10], cancer [11], chronic kidney disease [12] and obesity [13]. Evidence is now emerging that people with ID are disproportionally negatively impacted by COVID-19 [5 14 15]. The number of deaths of people with ID in England was three times higher in 2020 when compared to the corresponding period two years before [16] and people with ID may be more seriously affected by COVID-19 at a younger age than the general population [14 17]. Those with Down syndrome may be at particular risk [18 19].

Given the existing health inequalities for people with ID, it is reasonable to further examine how people with COVID-19 and ID present to and progress through the acute hospital system and how this compares to the experiences of the general population. To date, only a few small-scale studies have examined the clinical presentation of COVID-19 in people with ID [14 15] and none have provided a comprehensive picture of their experiences once admitted to hospital for COVID-19. Specifically, there is little evidence relating to resources and treatment allocation for people with ID and how this compares to the general population.

Decisions around escalation of care, for example to Intensive Care Units (ICU), are complicated during a pandemic with added pressures related to rationing of resources. Such decisions have come under increasing scrutiny during the COVID-19 [20 21]. In the UK the NHS offered guidance to hospital trusts related to resource allocation [22], however there is little research about the impact of these guidelines on vulnerable populations such as people with ID.

The aim of our study was to explore the hospital journey of patients with ID compared to the general population after they were admitted to hospital for COVID-19 during the first wave of the pandemic, when pressure on health care systems were most acute. We have chosen to focus on interventions that require triaging and resource allocation, for both clinical and supply reasons [22-24]: non-invasive ventilation (NIV), tracheal intubation and admission to ITU. Comparisons were made to the general population in the following areas: 1) pattern and severity of COVID-19 symptoms at time of hospital admission; 2) comorbidities; 3) admission to intensive care and use of intubation and/or ventilation treatments; 4) complications during hospital admission; 5) outcomes of admission including length of stay and mortality.

Method

For peer review only - http://bmjopen.bmj.com/site/about/guidelines.xhtml

Participants and study design

This study used data from The International Severe Acute Respiratory and emerging Infections Consortium (ISARIC) World Health Organization (WHO) Clinical Characterisation Protocol UK (CCP-UK). The ISARIC4C CCP-UK is an ongoing prospective cohort study in 260 hospitals across England, Scotland, and Wales of patients of any age who are hospitalised due to a confirmed or highly likely SARS-CoV-2 infection (National Institute for Health Research Clinical Research Network Central Portfolio Management System ID 14152). Our dataset consisted of patient demographic information, comorbidities, vital signs, COVID-19 related admission signs and symptoms, complications due to COVID-19, information regarding interventions and outcome of hospitalisation. Further information can be found online [8]. Overall in our sample were a total of 59,025 patients who were admitted between February 2020 and July 9, 2020 (downloaded on July 24, 2020). We identified 566 (0.96%) patients who had a diagnosis of ID and matched these patients to general population controls in the same dataset by age group, sex and ethnicity using a 1:3 ratio of ID patients to controls with no duplication of controls. Of the 566 ID patients, 506 had complete data on age group, sex and ethnicity and were matched to 1518 general population controls.

Patient and public involvement

Patients were not involved in the design, conduct, or reporting of this research as previous reported [8].

Statistical Analysis

Descriptive statistics were used to show patient information, comorbidities and COVID-19 admission information, medical complications, interventions and outcomes. Statistical testing was performed using fisher's exact test for frequency data while Mann Whitney U was used for respiratory rate on admission and linear regression for frailty scores adjusted for age group and sex.

We conducted logistic regression modelling to examine whether demographic variables (age group and sex), severity of COVID-19 illness on admission (respiratory rate, need for oxygen therapy and shortness of breath), the number of comorbidities patients had on admission, a diagnosis of Down syndrome or an ID diagnosis were associated with COVID-19 related interventions. Similar logistic regression models were used to examine factors associated with mortality between groups, and with medical complications due to COVID-19. In the mortality between groups model we adjusted for significant mortality related comorbidities for COVID-19 that have been previously reported in the ISARIC4C CCP-UK dataset; these included chronic cardiac disease, chronic pulmonary disease, chronic kidney disease, liver disease, obesity, chronic neurological disorder, dementia and malignant neoplasm [8]. We reported risk ratios (RRs) with 95% confidence intervals (CIs) for the logistic regression models. Time-to-event analysis using Cox proportional hazards modelling was used to examine how soon after admission patients with ID were dying from COVID-19 compared to controls while adjusting for covariates (age group, sex, severity of COVID-19 on admission, number of comorbidities and DS diagnosis). We used death as the outcome and data were depicted with a Kaplan-Meier curve. Finally, potential differences in length of stay between ID patients and controls

For peer review only

were explored using linear regression adjusting for the same covariates as the Cox proportional hazards model. To avoid violation of normality, clinical frailty scores and days in hospital was log-transformed and back transformed for reporting. All data analyses were done using R version 4.0.3.

Results

Description of study population and comorbidities

The sample of 506 ID patients consisted predominantly of adults over the age of 40 with only 25% of patients being under 40. Moreover, ID patients were mostly male and white, and had lower rates of chronic cardiac disease, hypertension, chronic pulmonary disease, asthma, malignant neoplasm, and rheumatologic disorders and were less likely to be smokers than the general population controls (table 1). On the other hand, higher rates of chronic neurological disorders (a broad category including cerebral palsy, multiple sclerosis, motor neurone disease, muscular dystrophy, myasthenia gravis, Parkinson's disease, stroke, severe learning difficulty) were reported in ID patients compared to controls, with a higher prevalence of dementia. The increased dementia rate is likely secondary to the association between Down syndrome and Alzheimer's disease included in the ID group.

For peer review only

		Controls		ID group		p value of comparison
		n	*N*	*n*	*N*	
		1518		506		
Age group (%)						
	<20	117 (7.7)		39 (7.7)		
	20-29	114 (7.5)		38 (7.5)		
	30-39	150 (9.9)		50 (9.9)		
	40-49	159 (10.5)		53 (10.5)		
	50-59	336 (22.1)		112 (22.1)		
	60-69	324 (21.3)		108 (21.3)		
	70-79	207 (13.6)		69 (13.6)		
	80+	111 (7.3)		37 (7.3)		
Sex (%)						
	Female	660 (43.5)		220 (43.5)		
	Male	858 (56.5)		286 (56.5)		
Ethnicity (%)						
	Aboriginal/First Nations	3 (0.2)		1 (0.2)		
	Black	36 (2.4)		12 (2.4)		
	East Asian	3 (0.2)		1 (0.2)		
	Other	96 (6.3)		32 (6.3)		
	South Asian	57 (3.8)		19 (3.8)		
	West Asian	9 (0.6)		3 (0.6)		
	White	1314 (86.6)		438 (86.6)		
Chronic cardiac disease		309 (21.5)	1439	81 (16.9)	479	**0.036**
Hypertension (physician diagnosed)		252 (31.9)	791	56 (18.7)	300	**<0.001**
Chronic pulmonary disease (not asthma)		191 (13.4)	1430	44 (9.2)	478	**0.016**

For peer review only

Asthma (physician diagnosed)	270 (18.8)	1435	68 (14.1)	481	**0.022**
Chronic kidney disease	140 (9.8)	1433	53 (11.0)	481	0.432
Mild, Moderate or severe liver disease[a]	54 (3.8)	1429	15 (3.1)	480	0.574
Diabetes[b]	266 (18.9)	1407	85 (18.2)	467	0.784
Chronic neurological disorder	156 (10.9)	1432	177 (36.6)	483	**<0.001**
Malignant neoplasm	100 (7.0)	1426	20 (4.2)	476	**0.029**
Chronic hematologic disease	39 (2.7)	1427	13 (2.7)	476	1.000
Obesity (as defined by clinical staff)	207 (15.7)	1317	69 (16.0)	431	0.879
Rheumatologic disorder	99 (6.9)	1426	20 (4.2)	473	**0.037**
Dementia	85 (5.9)	1437	47 (9.9)	473	**0.005**
Malnutrition	30 (2.2)	1378	12 (2.6)	459	0.590
Smoking					
Former Smoker	279 (26.4)	1055	43 (13.7)	313	**<0.001**
Never Smoked	676 (64.1)		247 (78.9)		
Yes	100 (9.5)		23 (7.3)		

The sample of 506 patients with an intellectual disability diagnosis from the UK ISARIC-4C matched to 1518 controls without an intellectual disability diagnosis based on age group, sex and ethnicity. [a]Mild, moderate and severe liver disease were combined into one category. [b]The variables diabetes and type, diabetes with complications, and diabetes without complications were combined into one category. The number of patients in the ID group with the comorbidities listed above on admission to hospital were compared to controls using Fisher's exact test.

Table 1. Characteristics of patients hospitalised for COVID-19 with and without an ID diagnosis

For peer review only

Signs, symptoms and severity of illness on admission in hospitalised COVID-19 patients with and without an ID diagnosis

A number of significant differences were observed in the symptoms at initial presentation to hospital between ID and control groups (Table 2). In particular, subjectively reported signs and symptoms such as loss of taste/smell, as well as those related to pain (headache, chest pain and muscle aches) were all reported less frequently in patients with ID. On the other hand, altered consciousness or confusion (29.9% vs 17.6%) and seizures (9.9% vs 2.2%) were more common in patients with ID. Compared to controls, ID patients were admitted with higher respiratory rates and were more likely to require oxygen therapy. In addition, adjusted for age group and sex, having a diagnosis of ID was significantly associated with higher clinical frailty scores.

	**Controls**		**ID group**		**p value of comparison**
	**n (%)**	**N**	**n (%)**	**N**	
Cough	972 (67.6)	1438	309 (64.6)	478	0.239
Cough with sputum production*	285 (22.7)	1254	58 (14.6)	397	**<0.001**
Cough with bloody sputum	41 (3.3)	1240	9 (2.3)	393	0.401
Fever	1004 (69.6)	1442	335 (69.8)	480	1.000
Sore throat	123 (10.4)	1186	29 (8.0)	364	0.191
Runny nose*	49 (4.2)	1168	6 (1.7)	357	**0.023**
Wheezing	94 (7.7)	1228	41 (10.1)	407	0.145
Ear pain	7 (0.6)	1150	3 (0.8)	364	0.711
Chest pain*	225 (17.8)	1267	35 (8.7)	404	**<0.001**
Muscle aches*	275 (23.1)	1192	30 (8.4)	357	**<0.001**
Joint pain	70 (6.1)	1147	18 (5.1)	356	0.520
Fatigue	511 (40.7)	1254	145 (37.5)	387	0.260
Shortness of breath*	953 (67.3)	1416	274 (59.8)	458	**0.004**
Disturbance or loss of taste*	51 (8.8)	578	3 (1.4)	207	**<0.001**
Disturbance or loss of smell*	36 (6.1)	588	1 (0.5)	212	**<0.001**
Headache*	177 (14.9)	1184	20 (5.5)	362	**<0.001**
Altered consciousness or confusion*	233 (17.6)	1326	124 (29.9)	415	**<0.001**
Seizures	28 (2.2)	1291	41 (9.9)	415	**<0.001**
Abdominal pain	187 (14.6)	1280	53 (13.2)	403	0.514
Vomiting and/or Nausea*	323 (24.3)	1329	67 (15.7)	426	**<0.001**
Diarrhoea*	279 (21.0)	1327	58 (13.4)	432	**<0.001**
Conjunctivitis	11 (0.9)	1205	4 (1.0)	384	0.767
Lymphadenopathy	10 (0.8)	1206	0 (0.0)	390	0.131
Skin rash	33 (2.7)	1228	8 (2.0)	396	0.581
Skin ulcers	19 (1.5)	1231	6 (1.5)	401	1.000
Haemorrhage	19 (1.5)	1261	4 (1.0)	416	0.626
Requirement of oxygen therapy on admission	406 (28.9)	1407	170 (35.1)	484	**0.011**

For peer review only

Median respiratory rate (breaths per minute) on admission (interquartile range)[a]	21 (10-50)	1404	22 (10-48)	464	**0.009**
Mean clinical frailty score (SD)	3.55 (2.17)	437	5.14 (1.89)	175	**<0.0001**

The number of patients in the ID group presenting with COVID-19 related symptoms on admission to hospital, compared to controls using Fisher's exact test. [a]We excluded respiratory rate values that were below 10 or higher than 50 breaths per minute as such data were considered outliers.

Table 2. Admission signs, symptoms and severity of illness on admission related to COVID-19 in hospitalised patients with and without an ID diagnosis

Medical complications among hospitalised COVID-19 patients with and without an ID diagnosis

In both the ID and general population groups the leading complications due to COVID-19 (Appendix Table 1) were pulmonary complications including viral pneumonia, bacterial pneumonia and acute respiratory syndrome, as well as acute renal injury and/or acute renal failure, anaemia and cardiac complications. Overall, medical complications were comparable between patients with ID and controls, with the exception of seizures which were more prevalent in the ID group (5.1% of those with ID compared to 2.0% of the control group).

Factors associated with COVID-19 related interventions

An increased likelihood of admission to ITU, tracheal intubation and non-invasive respiratory support were all associated with higher respiratory rate, shortness of breath and the requirement of oxygen therapy on admission, suggesting that the severity of illness on admission is important for prognosis and the need for COVID-19 related interventions. Significantly fewer ID patients were admitted to ITU, underwent intubation or received non-invasive respiratory support compared to controls (Table 3). Adjusted for age group, sex, severity of illness on admission, number of comorbidities and DS diagnosis, patients with ID were 37% less likely to receive non-invasive respiratory support, 40% less likely to receive intubation and 50% less likely to be admitted to the ICU while in hospital (Figure 1).

	**Controls**		**ID group**		**p value of**
	**n**	**N**	**n**	**N**	**comparison**
Non-invasive respiratory support	243 (16.9)	1436	60 (12.3)	487	**0.017**
Tracheal intubation	167 (11.2)	1496	36 (7.2)	503	**0.010**
Tracheostomy	16 (2.5)	637	2 (1.1)	178	0.390
Any time in intensive care unit	304 (20.3)	1500	59 (11.7)	505	**<0.001**

Table 3. COVID-19 related interventions for hospitalised patients with and without an intellectual disability diagnosis

For peer review only

Mortality rates and factors associated with mortality among COVID-19 patients with and without an ID diagnosis

People with ID had a 56% increased risk of dying from COVID-19 after they were hospitalised compared to controls, with a mortality rate of 29.2% for the ID group compared to 18.8% for controls (Appendix Figure 1.). Adjusted for age group, sex, known mortality related comorbidities, severity of illness on admission, interventions and DS diagnosis, the association between mortality and an ID diagnosis remained significant (Appendix Table 2).

Examining the factors associated with mortality in the ID group only we found that age (50 years and older), requiring oxygen therapy and higher respiratory rates at admission were all significantly associated with increased risk of dying from COVID-19. None of the known mortality-related comorbidities were significantly associated with mortality in patients with ID in our sample (Appendix Table 3).

Insert Figure 1 around here

Associations between medical complications and mortality

Viral pneumonia was significantly associated with mortality in the ID group. This complication increased ID patients' risk of dying by 174%. Acute respiratory syndrome was also strongly associated with mortality and increased ID patients' risk of dying by 107% (Appendix Table 4).

In comparison, while still significantly associated with mortality in controls, viral pneumonia was associated with a 56% increase in risk of dying and acute respiratory syndrome increased risk of dying by 91%. On the other hand, cardiac arrest was associated with a 438% increase risk of dying in controls, gastrointestinal haemorrhage increased the risk of dying by 178%, acute renal injury by 99% and other cardiac complications by 82% (Appendix Table 5).

Survival analysis of COVID -19 patients with and without an ID diagnosis

After five days in hospital, 16.6% of ID patients had died compared to only 6.5% of controls. This trend continued so that at twenty days 39.3% of ID patients had died compared to 32.7% of controls (Appendix Table 6). Figure 2 shows the Kaplan-Meier estimates of survival probability for our ID group and controls. Adjusting for age group, sex, DS diagnosis, number of comorbidities and severity of COVID-19 on admission, the hazard ratio (HR) for COVID-19 related mortality in patients with ID compared to controls was 1.44 (95% CI = 1.13 - 1.84, p = 0.003**).** Therefore, patients with ID were dying 1.44 times faster than controls at any particular point in time after they were admitted to hospital for COVID-19, even after adjusting for covariates.

Insert Figure 2 around here

Factors associated with length of time in hospital for COVID-19 patients with and without an ID diagnosis

For peer review only

A significant association between a diagnosis of ID and length of time in hospital was found, with ID patients spending longer periods in hospital after they were admitted for COVID-19. The controls spent a mean of 10.98 days in hospital (SD = 14.45, median = 6.5 days) while the ID group spent 14.55 days on average (SD = 13.29, median = 11 days; Appendix Figure 2). Other factors significantly associated with longer stays in hospital in both groups were being older than 20 years, more comorbidities and greater severity of illness on admission.

	**exp(β)**	**95% CI**	**p value**
Age group			
20-29 years old	1.23	1.10 - 1.37	**0.0002**
30-39 years old	1.30	1.17 - 1.43	**<0.0001**
40-49 years old	1.36	1.23 - 1.50	**<0.0001**
50-59 years old	1.40	1.28 - 1.54	**<0.0001**
60-69 years old	1.46	1.33 - 1.61	**<0.0001**
70-79 years old	1.48	1.34 - 1.65	**<0.0001**
80+ years old	1.69	1.49 - 1.92	**<0.0001**
Male sex	1.03	0.98 - 1.07	0.240
Shortness of breath	0.96	0.91 - 1.01	0.107
Respiratory rate	1.01	1.00 - 1.01	**0.0003**
No oxygen therapy	0.91	0.86 - 0.95	**<0.0001**
Number of comorbidities	1.05	1.04 - 1.07	**<0.0001**
DS diagnosis	1.08	0.95 - 1.22	0.229
ID diagnosis	1.15	1.09 - 1.22	**<0.0001**

Table 4. Factors associated with hospital length of stay in COVID-19 patients

DISCUSSION

This is the first in-depth exploration of treatment and interventions offered to patients with ID who were admitted to hospital for COVID-19, which found that the hospital journey for people with ID and COVID-19 is substantially different to the general population in a number of fundamental areas: recognition and assessment of COVID-19 symptoms; symptoms and severity of illness on admission; access to interventions and ICUs; mortality rates, survival trajectories and duration of hospital stay.

Recognition and Assessment of COVID-19 Symptoms

The most prevalent symptoms recorded at admission in both the ID and control group were cough, fever and shortness of breath, in keeping with previous reports [25]. However, patients with ID were significantly less likely to present with subjective symptoms including pain, loss of taste or smell, and 'shortness of breath', despite having higher respiratory rates at admission, while being more likely to present with altered consciousness, confusion and seizures, which could indicate a more severe presentation upon admission. Patients with ID also presented with other indicators of more severe illness at the point of admission, including greater requirement for supplemental oxygen therapy and increased average respiratory rates compared to controls. There are several potential explanations for late presentation of

For peer review only

patients with ID: poor symptom recognition by caregivers and patients themselves, communication difficulties, and exclusion from digital information and public health campaigns which could reduce awareness about early warning signs and symptoms.

Course of illness in hospitalised patients with ID and access to Interventions and Intensive Care Units

Once admitted, patients with ID and COVID-19 had a more aggressive course of disease, with higher rates of death in the early stages of hospitalisation as well as longer hospital stays. Rates of complications and most comorbidities were comparable between the groups, however patients with ID were given higher scores on the clinical frailty scale, potentially reflecting misinterpretation of the degree of frailty in the context of long-term but stable cognitive impairment. This has implications for treatment decisions around resource allocation when availability may be limited.

Despite having more severe symptoms upon admission and similar rates of complications, patients with ID were less likely to be treated with non-invasive ventilation, tracheal intubation, or be admitted to an ICU setting. This disparity in access to appropriate treatment has been highlighted in investigations of other conditions [26], with issues surrounding decision-making capacity, ceilings of care, inappropriate use of clinical frailty scales, and discrimination or biases potentially contributing to inequalities in care [27]. Other contributing factors may be related to tolerability of interventions (particularly NiV) for people with ID, perceived treatment difficulties that may influence decision making, and inappropriate use of Do Not Resuscitate orders (DNaCPRs).

Complications of COVID-19 Infection, Mortality Rates, and Length of Stay

Having a diagnosis of ID was associated with a 56% increase in mortality risk, which was not associated with seizures or dementia, despite these conditions being more common in ID patients compared to the general population, particularly those with Down syndrome [28]. The increased mortality also does not appear to be related to other suggested COVID-19 comorbidities for adverse outcome [8 10 12], although as in the general population, older age and severity of illness on admission did show significant associations with mortality in ID. As well as an increased mortality rate in ID patients after admission to hospital, we found a different survival trajectory. ID patients died at a 1.44 times faster rate than the general population, even when age, comorbidities and severity of symptoms were considered. This suggests that aspects of their care and treatment may be contributing to increased mortality rather than co-morbidities or complications.

People with ID who survived had a longer inpatient stay on average. Again, this does not appear to be secondary to increased complications or co-morbidities. It is therefore possible that people with ID may be experiencing delays in their discharge and support to return to the community. Longer admissions can be associated with distress for the individual, exposure to risk of hospital acquired infections, and institutionalisation. These findings highlight the different experiences of patients with ID after they were admitted to hospital for COVID-19 compared to the general population.

For peer review only

Strengths and limitations

The strengths of the study are the large sample size and the use of a well-matched control group which allows for comparisons in symptoms, treatment and outcomes to be captured. Data was taken from across the UK meaning it is reflective of experiences across the country rather than regionally specific issues. It used real-world data captured during an acute and evolving pandemic and gives insight into conditions faced by patients and health professionals at the time.

Some limitations are acknowledged. The study relied upon data captured at the time of care. Whilst this provides an accurate picture of acute clinical care, the nature of clinical records can lead to some degree of missing or incomplete data. In addition, the use of combined group categories (particularly the heterogenous group "chronic neurological disorder") limited the ability to explore the potential impact of specific diseases, while the reason for specific clinical decisions may not be clear. Further research is therefore needed to explore the details around clinical decision making for people with ID during pandemic conditions and the impact of care rationalisation on this population. It will also be important to understand the experiences of individuals with ID and their caregivers, particularly with regards to decision making, advocacy and inclusion.

Conclusion

These findings highlight an ongoing disparity in healthcare between people with ID and the general population which have been magnified by the COVID-19 pandemic, with implications for improving care and treatment during the ongoing crisis to ensure the levelling-up of services for the future. It is hard not to be concerned at the possibility of bias and discrimination affecting treatment decisions in such conditions, whether implicit or explicit. Barriers to care will need to be overcome and information should be disseminated in an accessible way to both caregivers and people with ID, particularly with regards to early symptoms and warning signs of a more severe presentation. In the community digital exclusion has been identified as a barrier to information for people with ID [29]. This may make it more difficult for people with ID to report early signs, receive up to date information about risks, or indeed even be part of track-and-trace systems. They may also be less able to self-monitor for early signs such as fevers. Moves towards the use of home oxygen saturation monitoring may be helpful in this population in identifying at risk people before they become acutely unwell and could allow for treatment to be initiated in a timely manner to reduce mortality.

Similarly, the results stress the need for people with ID admitted for COVID-19 (and other similar infections) to be prioritised for enhanced care and monitoring based on indicators of deterioration, without reliance on self-reporting. Earlier intervention may be indicated to avoid the more aggressive course of illness. Provisions and training should be in place in all hospitals regarding capacity and decision making, and trained staff should be available to assist in these matters. Care should be taken when making decisions about prioritisation of interventions to ensure they are not biased against people with long-term disabilities, but instead based on relevant prognostic indicators. Medical ethics panels which include

For peer review only

professionals who are familiar with the care and needs of people with ID could assist with such decisions.

It is hoped that these results from the first wave of the pandemic highlight the ongoing health disparities faced by people with ID and will help raise awareness and mobilise health care services to improve practices and access for this population.

Data availability statement

Researchers wishing to access the data can contact the MRC funded ISARIC 4C Consortium.

Author Contributions

AS conceived and designed the project with help from RAB. RAB and AS planned the data analysis. RAB conducted the data analysis. RAB, SP and JS wrote the first draft with input from AS. All authors contributed to reviewing and revising the manuscript and agreed final approval of the version to be published.

Acknowledgements and funding

This report is independent research which used data provided by the MRC funded ISARIC 4C Consortium and which the Consortium collected under a research contract funded by the National Institute for Health Research. The views expressed in this publication are those of the authors and not necessarily those of the ISARIC 4C consortium.

This study was supported by grants from the MRC (MR/S011277/1; MR/S005145/1; MR/R024901/1), the European Commission (H2020 SC1 Gene overdosage and comorbidities during the early lifetime in Down Syndrome GO-DS21- 848077) and Alzheimer's Society (AS-CP-18-0020: fellowship to SP).

This study represents independent research part funded by the National Institute for Health Research (NIHR) Maudsley Biomedical Research Centre at South London and Maudsley NHS Foundation Trust and King's College London. The views expressed are those of the authors and not necessarily those of the NHS, the NIHR or the Department of Health and Social Care.

For peer review only

REFERENCES

1. Maulik PK, Mascarenhas MN, Mathers CD, et al. Prevalence of intellectual disability: a meta-analysis of population-based studies. *Research in developmental disabilities* 2011;32(2):419-36.
2. Heslop P, Blair PS, Fleming P, et al. The Confidential Inquiry into premature deaths of people with intellectual disabilities in the UK: a population-based study. *The Lancet* 2014;383(9920):889-95.
3. Cooper S-A, Allan L, Greenlaw N, et al. Rates, causes, place and predictors of mortality in adults with intellectual disabilities with and without Down syndrome: cohort study with record linkage. *BMJ Open* 2020;10(5):e036465. doi: 10.1136/bmjopen-2019-036465

4. Cuypers M, Schalk B, Koks-Leensen M, et al. Mortality of people with intellectual disabilities during the 2017/2018 influenza epidemic in the Netherlands: potential implications for the COVID-19 pandemic. *Journal of Intellectual Disability Research* 2020;64(7):482-88.
5. Landes SD, Turk MA, Formica MK, et al. COVID-19 outcomes among people with intellectual and developmental disability living in residential group homes in New York State. *Disability and Health Journal* 2020;13(4):100969.
6. Amon JJ, Wurth M. A Virtual Roundtable on COVID-19 and Human Rights with Human Rights Watch Researchers. *Health Hum Rights* 2020;22(1):399-413.
7. Dong E, Du H, Gardner L. An interactive web-based dashboard to track COVID-19 in real time. *The Lancet Infectious Diseases* 2020;20(5):533-34. doi: https://doi.org/10.1016/S1473-3099(20)30120-1
8. Docherty AB, Harrison EM, Green CA, et al. Features of 20 133 UK patients in hospital with covid-19 using the ISARIC WHO Clinical Characterisation Protocol: prospective observational cohort study. *bmj* 2020;369
9. Verity R, Okell LC, Dorigatti I, et al. Estimates of the severity of coronavirus disease 2019: a model-based analysis. *The Lancet infectious diseases* 2020;20(6):669-77.
10. Yang J, Zheng Y, Gou X, et al. Prevalence of comorbidities and its effects in patients infected with SARS-CoV-2: a systematic review and meta-analysis. *International Journal of Infectious Diseases* 2020;94:91-95.
11. Fung M, Babik JM. COVID-19 in immunocompromised hosts: what we know so far. *Clinical Infectious Diseases* 2021;72(2):340-50.
12. Richardson S, Hirsch JS, Narasimhan M, et al. Presenting characteristics, comorbidities, and outcomes among 5700 patients hospitalized with COVID-19 in the New York City area. *Jama* 2020;323(20):2052-59.
13. Simonnet A, Chetboun M, Poissy J, et al. High prevalence of obesity in severe acute respiratory syndrome coronavirus-2 (SARS-CoV-2) requiring invasive mechanical ventilation. *Obesity* 2020;28(7):1195-99.
14. Perera B, Laugharne R, Henley W, et al. COVID-19 deaths in people with intellectual disability in the UK and Ireland: descriptive study. *BJPsych open* 2020;6(6)
15. Turk MA, Landes SD, Formica MK, et al. Intellectual and developmental disability and COVID-19 case-fatality trends: TriNetX analysis. *Disability and Health Journal* 2020;13(3):100942.
16. Public Health England. Deaths of people identified as having learning disability with COVID-19 in England in the spring of 2020. https://assets.publishing.service.gov.uk/government/uploads/system/uploads/attachment_data/file/933612/COVID-19__learning_disabilities_mortality_report.pdf 2020.
17. Care Quality Commission. CQC publishes data on deaths of people with a learning disability: Care Quality Commission; 2020 [Available from: https://www.cqc.org.uk/news/stories/cqc-publishes-data-deaths-people-learning-disability accessed 24 March 2021 2021.
18. Hüls A, Costa AC, Dierssen M, et al. Medical vulnerability of individuals with Down syndrome to severe COVID-19–data from the Trisomy 21 Research Society and the UK ISARIC4C survey. *EClinicalMedicine* 2021;33:100769.
19. Illouz T, Biragyn A, Frenkel-Morgenstern M, et al. Specific Susceptibility to COVID-19 in Adults with Down Syndrome. *Neuromolecular medicine* 2021:1-11.

For peer review only

20. Griffiths F, Svantesson M, Bassford C, et al. Decision-making around admission to intensive care in the UK pre-COVID-19: a multicentre ethnographic study. *Anaesthesia* 2020;76(4) doi: 10.1111/anae.15272
21. Montgomery J, Stokes-Lampard H, Griffiths M, et al. Assessing whether COVID-19 patients will benefit from critical care, and an objective approach to capacity challenges during a pandemic: an intensive care Society clinical guideline. *Journal of the Intensive Care Society* 2020;0(0):1-7.
22. I.C.M. Anaesthesia. Clinical Guide for the Management of Critical Care for Adults with COVID-19 During the Coronavirus Pandemic. https://icmanaesthesiacovid-19.org/clinical-guide-for-the-management-of-critical-care-for-adults-with-covid-19-during-the-coronavirus-pandemic: ICM Anaesthesia Covid-19, 2020.
23. Phua J, Weng L, Ling L, et al. Intensive care management of coronavirus disease 2019 (COVID-19): challenges and recommendations. *The Lancet Respiratory Medicine* 2020;8(5):506-17.
24. White DB, Lo B. A framework for rationing ventilators and critical care beds during the COVID-19 pandemic. *Jama* 2020;323(18):1773-74.
25. Stokes EK, Zambrano LD, Anderson KN, et al. Coronavirus disease 2019 case surveillance—United States, January 22–May 30, 2020. *Morbidity and Mortality Weekly Report* 2020;69(24):759.
26. LeDeR. Deaths of people with learning disabilities from COVID-19. http://www.bristol.ac.uk/sps/leder/news/2020/leder-covid-19-reviews.html, 2020.
27. Ali A, Scior K, Ratti V, et al. Discrimination and other barriers to accessing health care: perspectives of patients with mild and moderate intellectual disability and their carers. *PloS one* 2013;8(8):e70855.
28. Firth NC, Startin CM, Hithersay R, et al. Aging related cognitive changes associated with Alzheimer's disease in Down syndrome. *Annals of clinical and translational neurology* 2018;5(6):741-51.
29. Caton S, Chapman M. The use of social media and people with intellectual disability: A systematic review and thematic analysis. *Journal of Intellectual & Developmental Disability* 2016;41(2):125-39. doi: 10.3109/13668250.2016.1153052

Figures title and caption:

Figure 1. Factors associated with interventions (non-invasive respiratory support, intubation and ICU) in hospitalised COVID-19 patients with and without an intellectual disability diagnosis

A: *Factors associated with access to non-invasive respiratory support (e.g. BIPAP, CPAP).* ***B:*** *Factors associated with the use of tracheal intubation.* ***C****: Factors associated with admission to ICU. Bold labels on the forest plots indicate statistically significant associations. Percent relative effects can be calculated using (RR - 1) x 100 for RRs over 1 or (1 - RR) x 100 for RRs less than 1. For example, shortness of breath on admission was associated with a 73% [(1.73-1) x 100] increase in risk of being admitted to the ICU while not requiring oxygen therapy of admission was associated with a 48% [(1-0.52) x 100] decrease in risk of being admitted to the ICU while in hospital. We present log-transformed RRs in the plots.*

Figure 2. Kaplan-Meier survival plot of hospitalised COVID-19 patients with and without an intellectual disability diagnosis

For peer review only

A

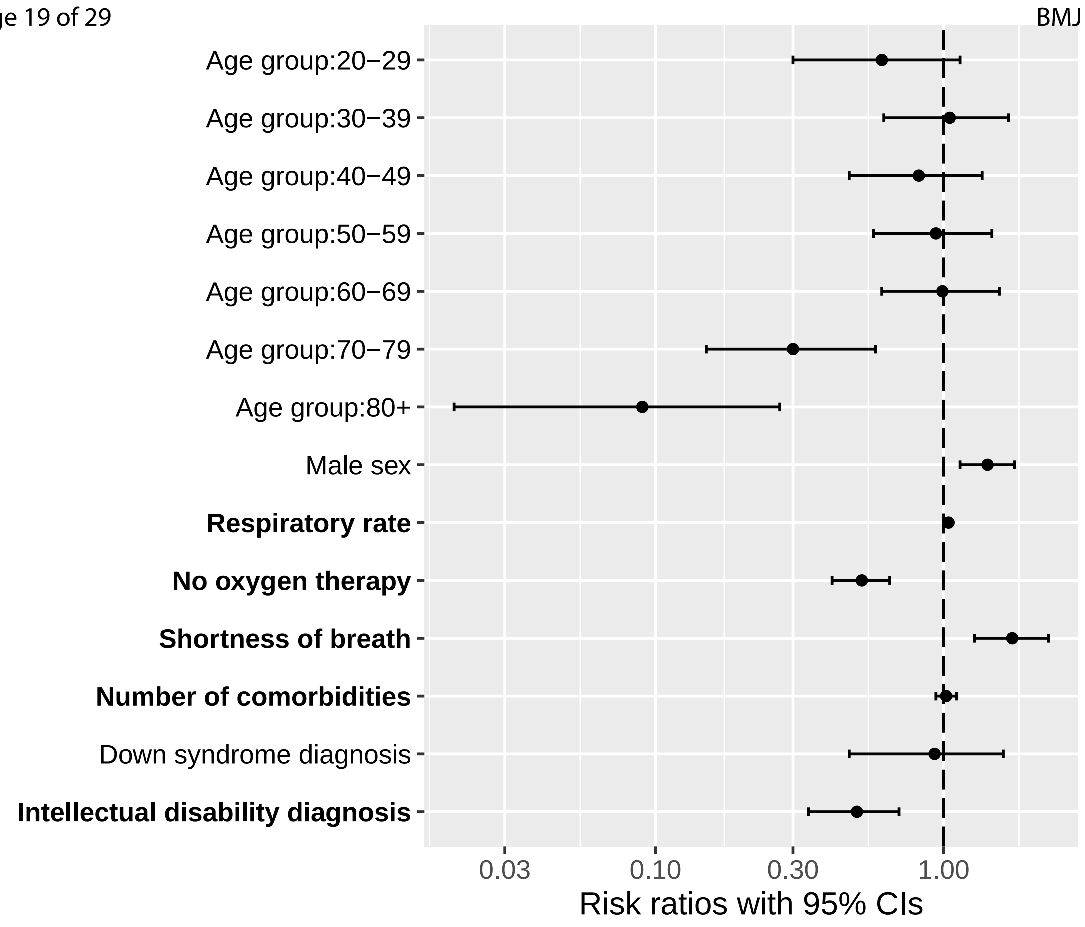

	Risk ratio	Lower CI	Upper CI	p value
Age group:20−29	1.13	0.48	2.47	0.771
Age group:30−39	1.31	0.63	2.62	0.474
Age group:40−49	1.33	0.65	2.63	0.435
Age group:50−59	1.73	0.93	3.18	0.095
Age group:60−69	1.79	0.96	3.27	0.079
Age group:70−79	0.83	0.39	1.77	0.630
Age group:80+	0.50	0.19	1.26	0.147
Male sex	1.27	1.00	1.59	0.053
Respiratory rate	1.04	1.02	1.06	<0.0001
No oxygen therapy	0.56	0.43	0.70	<0.0001
Shortness of breath	2.10	1.50	2.93	<0.0001
Number of comorbidities	1.14	1.05	1.24	0.002
Down syndrome diagnosis	1.38	0.76	2.22	0.261
Intellectual disability diagnosis	0.63	0.43	0.87	0.007

B

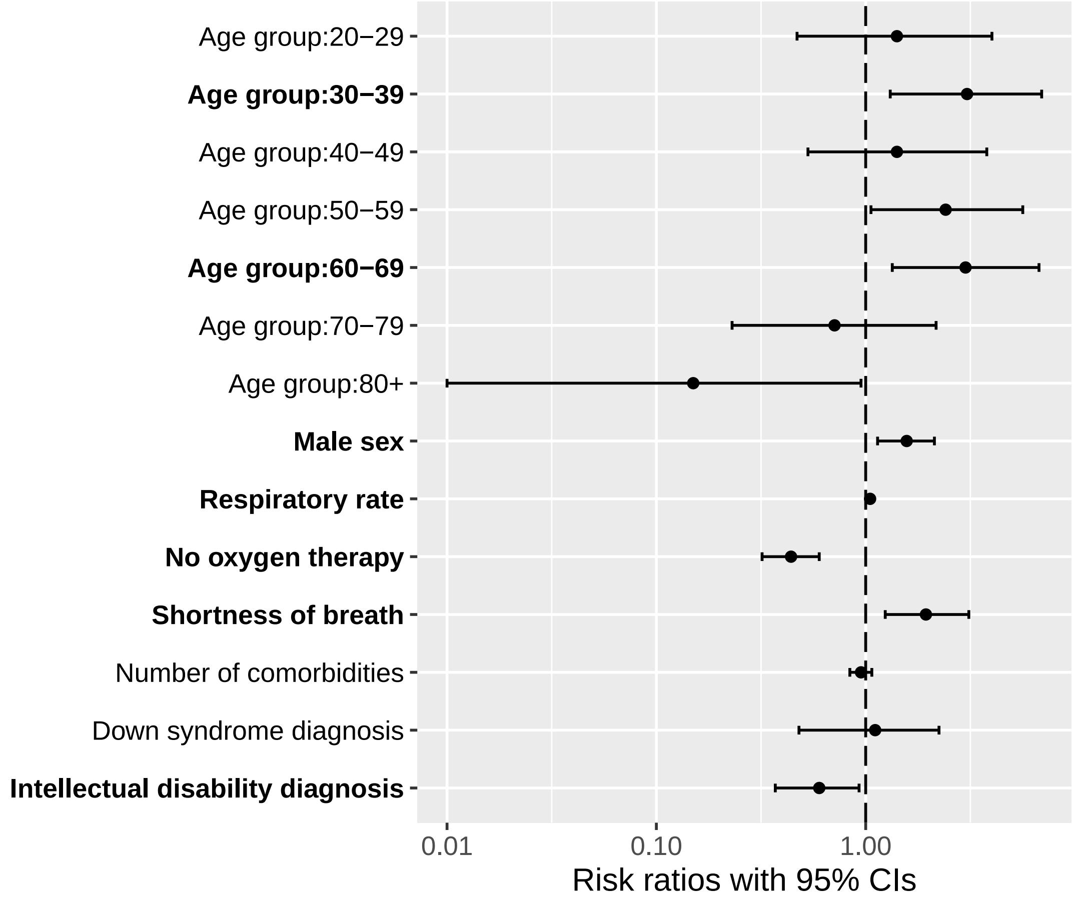

	Risk ratio	Lower CI	Upper CI	p value
Age group:20−29	1.41	0.47	4.01	0.536
Age group:30−39	3.05	1.31	6.93	0.015
Age group:40−49	1.41	0.53	3.79	0.499
Age group:50−59	2.41	1.06	5.63	0.051
Age group:60−69	3.00	1.34	6.73	0.014
Age group:70−79	0.71	0.23	2.17	0.538
Age group:80+	0.15	0.01	0.95	0.086
Male sex	1.57	1.14	2.13	0.006
Respiratory rate	1.05	1.03	1.07	<0.001
No oxygen therapy	0.44	0.32	0.60	<0.0001
Shortness of breath	1.94	1.24	3.11	0.006
Number of comorbidities	0.95	0.84	1.07	0.396
Down syndrome diagnosis	1.11	0.48	2.24	0.795
Intellectual disability diagnosis	0.60	0.37	0.93	0.028

C

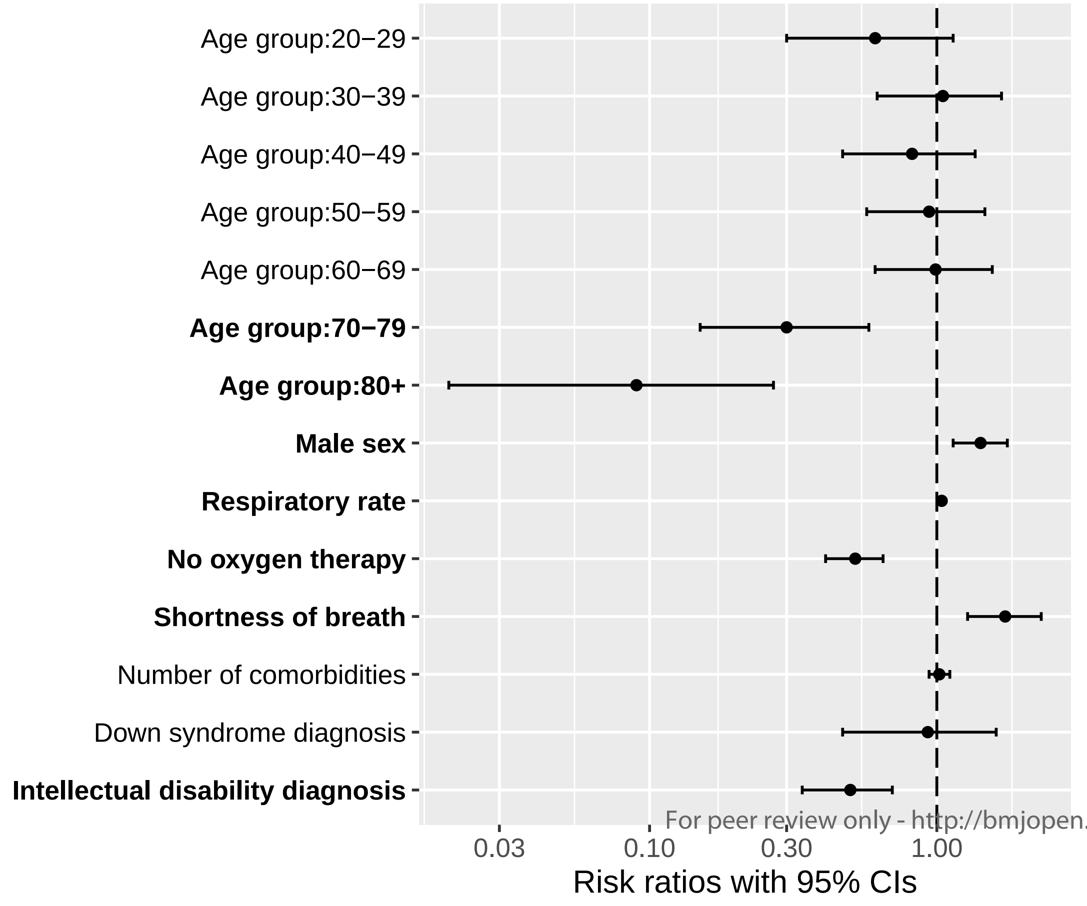

	Risk ratio	Lower CI	Upper CI	p value
Age group:20−29	0.61	0.30	1.14	0.131
Age group:30−39	1.05	0.62	1.68	0.852
Age group:40−49	0.82	0.47	1.36	0.454
Age group:50−59	0.94	0.57	1.47	0.786
Age group:60−69	0.99	0.61	1.56	0.982
Age group:70−79	0.30	0.15	0.58	0.0002
Age group:80+	0.09	0.02	0.27	<0.0001
Male sex	1.42	1.14	1.76	0.002
Respiratory rate	1.04	1.03	1.06	<0.0001
No oxygen therapy	0.52	0.41	0.65	<0.0001
Shortness of breath	1.73	1.28	2.31	0.0005
Number of comorbidities	1.02	0.94	1.11	0.584
Down syndrome diagnosis	0.93	0.47	1.61	0.810
Intellectual disability diagnosis	0.50	0.34	0.70	<0.0001

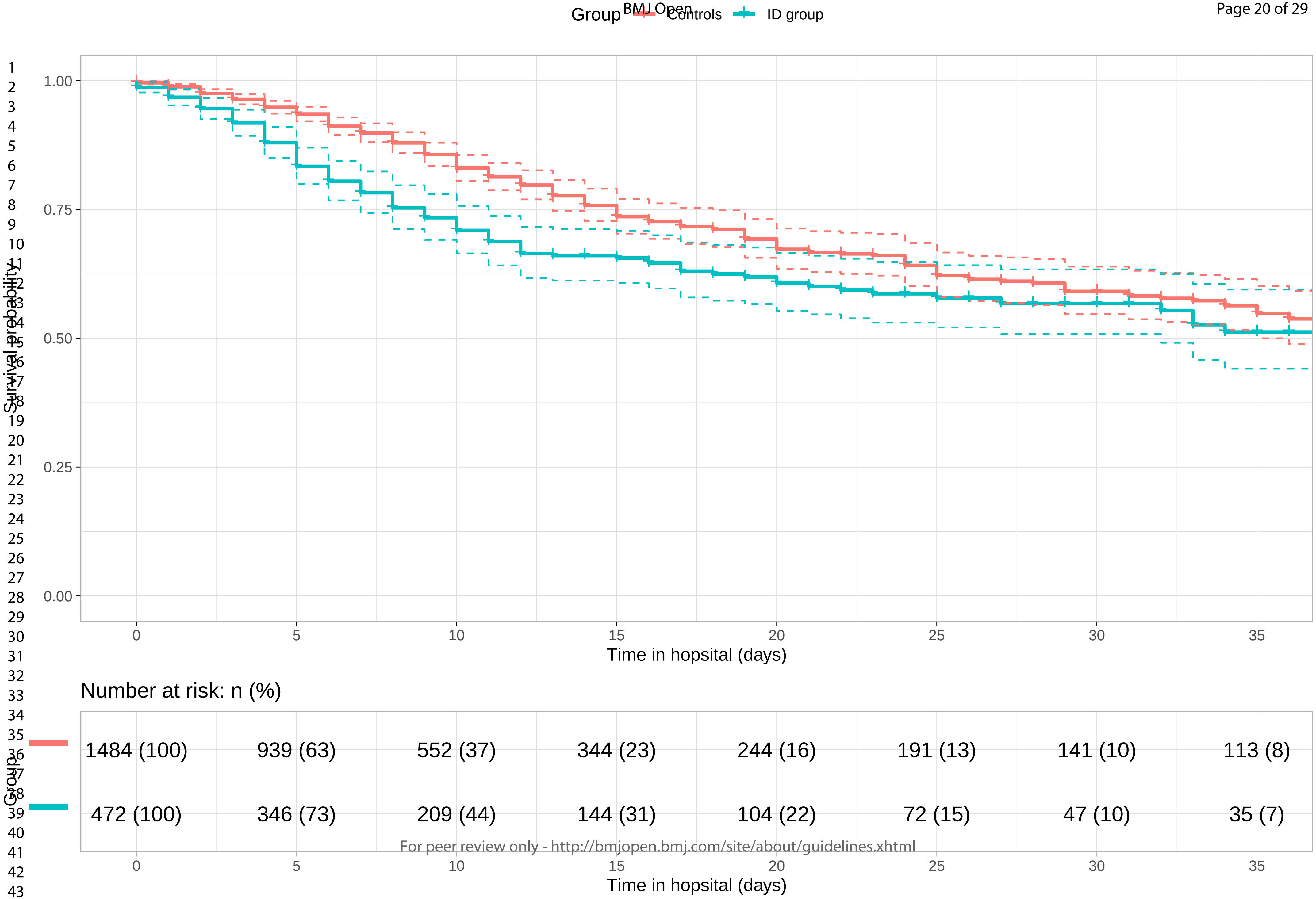

For peer review only - http://bmjopen.bmj.com/site/about/guidelines.xhtml

Appendix Table 1. Complications related to COVID-19 in hospitalised patients with and without an ID diagnosis

	Controls		ID group		p value of comparison
	n (%)	N	n (%)	N	
Viral pneumonia	595 (43.3)	1375	222 (47.4)	468	0.119
Bacterial pneumonia	153 (11.2)	1364	63 (13.6)	463	0.182
Acute respiratory syndrome	160 (11.6)	1382	44 (9.3)	471	0.201
Other lung complications[1]	97 (6.9)	1408	28 (5.9)	476	0.523
Meningitis / Encephalitis	6 (0.4)	1396	1 (0.2)	474	0.687
Seizures	28 (2.0)	1401	24 (5.1)	474	**0.001**
Other neurological complications[2]	31 (2.2)	1401	7 (1.5)	472	0.450
Cardiac arrest	31 (2.2)	1397	9 (1.9)	473	0.854
Other cardiac complications[3]	132 (9.4)	1409	34 (7.2)	473	0.160
Bacteraemia	40 (2.9)	1391	10 (2.1)	469	0.509
Gastrointestinal hemorrhage or coagulation disorder	44 (3.1)	1402	16 (3.4)	473	0.764
Pancreatitis	10 (0.7)	1395	1 (0.2)	473	0.309
Rhabdomyolysis / Myositis	5 (0.4)	1395	2 (0.4)	473	1.000
Anaemia	164 (11.7)	1404	46 (9.7)	473	0.273
Acute renal injury and/or acute renal failure	192 (13.7)	1402	57 (12.0)	475	0.389
Liver dysfunction	60 (4.3)	1396	16 (3.4)	472	0.422
Hypoglycaemia or hyperglycaemia	88 (6.3)	1386	30 (6.3)	473	1.000

[1]Combined cryptogenic organizing pneumonia (COP), pneumothorax, pleural effusion and bronchiolitis, [2]Combined Stroke / Cerebrovascular accident and other neurological complication, [3]Combined congestive heart failure, endocarditis / myocarditis pericarditis, myocarditis / pericarditis, cardiomyopathy, cardiac arrhythmia, cardiac ischemia. The number of patients in the ID group developing Covid-19 related complications while in hospital were compared to controls using Fisher's exact test.

For peer review only

Appendix Figure 1. Mortality rates of hospitalised COVID-19 patients with and without an intellectual disability diagnosis

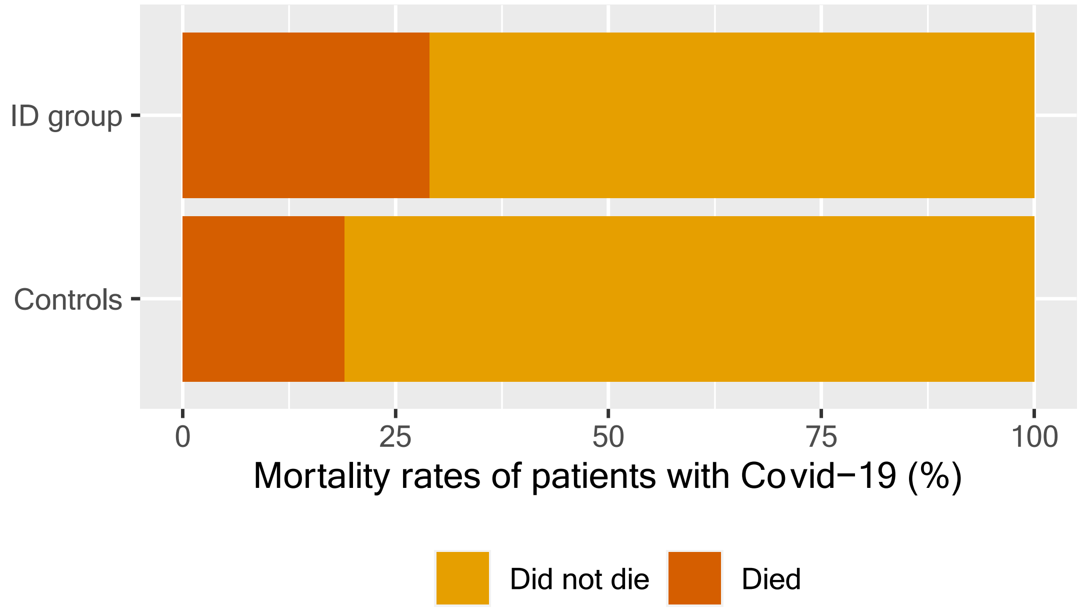

For peer review only

Appendix Table 2. Factors associated with mortality in hospitalised COVID-19 patients

	Risk ratio	95% CI	p value
Age group			
20-29 years old	3.39	0.40 - 44.51	0.300
30-39 years old	6.95	1.29 - 61.92	*0.064*
40-49 years old	10.17	2.06 - 72.36	**0.023**
50-59 years old	22.22	5.44 - 90.88	**0.001**
60-69 years old	25.94	6.57 - 93.93	**0.0006**
70-79 years old	37.26	10.31 - 100.09	**0.0001**
80+ years old	60.18	20.83 - 106.13	**<0.0001**
Male sex	1.18	0.918 - 1.50	0.191
Chronic cardiac disease	1.35	1.02 - 1.76	**0.038**
Chronic pulmonary disease	1.66	1.22 - 2.16	**0.002**
Chronic kidney disease	1.58	1.13 - 2.12	**0.009**
Liver disease	1.69	0.97 - 2.54	*0.055*
Obesity	1.21	0.97 - 1.62	0.242
Chronic neurological disorder	1.64	1.23 - 2.11	**0.001**
Dementia	1.11	0.71 - 1.64	0.632
Malignant neoplasm	1.32	0.84 - 1.92	0.209
Shortness of breath	0.96	0.70 - 1.29	0.785
Respiratory rate	1.03	1.02 - 1.05	**0.0003**
No oxygen therapy	0.72	0.55 - 0.91	**0.005**
Admission to ICU	0.92	0.51 - 1.51	0.758
Intubation	3.11	2.22 - 3.98	**<0.0001**
Non-invasive respiratory support	1.44	1.02 - 1.95	**0.039**
DS diagnosis	1.92	1.19 - 2.76	**0.009**
ID diagnosis	1.56	1.17 - 2.02	**0.003**

ICU, Intensive Care Unit; DS, Down syndrome, ID; Intellectual disability. Tracheostomy was not included in the model due to a large proportion of missing data.

For peer review only

Appendix Table 3. Factors associated with mortality in hospitalised COVID-19 patients with an intellectual disability diagnosis

	Risk ratio	95% CI	p value
Age group			
20-29 years old	1.12	0.13 - 4.99	0.903
30-39 years old	2.41	0.61 - 6.58	0.213
40-49 years old	2.85	0.80 – 7.00	0.120
50-59 years old	4.28	1.62 - 8.04	**0.012**
60-69 years old	6.43	3.21 - 9.07	**0.0002**
70-79 years old	4.04	1.40 - 7.93	**0.022**
80+ years old	7.33	3.74 - 9.44	**0.0001**
Male sex	1.24	0.84 - 1.71	0.267
DS diagnosis	1.41	0.86 - 2.02	0.152
Chronic cardiac disease	1.50	0.94 - 2.12	*0.085*
Chronic pulmonary disease	1.08	0.55 - 1.78	0.789
Chronic kidney disease	1.50	0.85 - 2.22	0.142
Liver disease	1.07	0.33 - 2.20	0.894
Obesity	0.93	0.48 - 1.52	0.803
Chronic neurological disorder	1.39	0.94 - 1.90	*0.091*
Dementia	1.25	0.66 - 2.01	0.454
Malignant neoplasm	0.81	0.28 - 1.69	0.633
Shortness of breath	0.99	0.62 - 1.47	0.960
Respiratory rate	1.02	1.00 - 1.05	**0.036**
No oxygen therapy	0.49	0.31 - 0.73	**0.0002**
Access to any intervention	1.54	0.99 - 2.15	*0.054*

For peer review only

Appendix Table 4. Associations between complications due to COVID-19 and mortality in patients with an ID diagnosis

	Risk ratio	95% CI	*p value*
Age group			
20-29 years old	2.39	0.22 - 22.71	0.478
30-39 years old	4.78	0.79 - 27.72	0.141
40-49 years old	7.00	1.39 - 30.29	*0.053*
50-59 years old	7.92	1.77 - 30.94	**0.032**
60-69 years old	14.97	4.16 -34.30	**0.003**
70-79 years old	7.37	1.52 - 30.59	**0.045**
80+ years old	18.11	5.11 -35.12	**0.001**
Male sex	1.11	0.77 - 1.54	0.550
Viral pneumonia	2.74	1.97 - 3.60	**<0.0001**
Bacterial pneumonia	1.60	0.98 - 2.29	*0.054*
Acute respiratory syndrome	2.07	1.28 - 2.88	**0.006**
Other lung complications[1]	1.76	0.93 -2.	*0.077*
Seizures	0.39	0.06 - 1.16	0.146
Other neurological complications[2]	0.87	0.15 - 2.50	0.844
Other cardiac complications[3]	0.64	0.24 -1.34	0.278
Bacteraemia	1.57	0.42 - 3.02	0.432
Gastrointestinal hemorrhage or coagulation disorder	0.51	0.12 -1.48	0.267
Anaemia	0.51	0.20 - 1.08	*0.096*
Acute renal injury / Acute renal failure	1.17	0.62 - 1.92	0.594
Liver dysfunction	0.90	0.21 - 2.11	0.851
Hypoglycaemia or hyperglycaemia	0.53	0.17 -1.25	0.183

[1]Combined cryptogenic organizing pneumonia (COP), pneumothorax, pleural effusion and bronchiolitis, [2]Combined Stroke / Cerebrovascular accident and other neurological complication, [3]Combined congestive heart failure, endocarditis / myocarditis pericarditis, myocarditis / pericarditis, cardiomyopathy, cardiac arrhythmia, cardiac ischemia. Meningitis, pancreatitis and rhabdomyolysis where removed from the model because they were recorded in less than 1% of ID patients. Ethnicity and cardiac arrest were also removed because they were not good predictors in the model.

For peer review only

Appendix Table 5. Associations between complications due to COVID-19 and mortality in patients without an ID diagnosis

	Risk ratio	95% CI	*p value*
Age group			
20-29 years old	1.07	0.042 - 22.07	0.961
30-39 years old	2.04	0.30 - 29.80	0.522
40-49 years old	4.12	0.75 - 45.82	0.179
50-59 years old	10.34	2.34 - 69.97	**0.018**
60-69 years old	13.12	3.05 - 76.02	**0.008**
70-79 years old	26.96	7.05 -91.13	**0.0005**
80+ years old	36.49	10.18 - 95.93	**0.0001**
Male sex	1.02	0.75 - 1.35	0.913
Viral pneumonia	1.56	1.16 - 2.07	**0.003**
Bacterial pneumonia	1.01	0.63 - 1.52	0.970
Acute respiratory syndrome	1.91	1.32 - 2.62	**0.0008**
Other lung complications[1]	1.11	0.64 - 1.79	0.683
Seizures	0.97	0.32 - 2.23	0.958
Other neurological complications[2]	0.93	0.33 - 2.06	0.881
Cardiac arrest	5.38	3.94 - 6.15	**<0.0001**
Other cardiac complications[3]	1.82	1.22 - 2.57	**0.004**
Bacteraemia	0.82	0.32 - 1.75	0.646
Gastrointestinal hemorrhage or coagulation disorder	2.78	1.60 - 4.09	**0.0009**
Anaemia	1.23	0.80 - 1.80	0.316
Acute renal injury / Acute renal failure	1.99	1.41 - 2.69	**0.0002**
Liver dysfunction	0.50	0.21 - 1.03	*0.072*
Hypoglycaemia or hyperglycaemia	1.15	0.64 - 1.90	0.620

[1]Combined cryptogenic organizing pneumonia (COP), pneumothorax, pleural effusion and bronchiolitis, [2]Combined Stroke / Cerebrovascular accident and other neurological complication, [3]Combined congestive heart failure, endocarditis / myocarditis pericarditis, myocarditis / pericarditis, cardiomyopathy, cardiac arrhythmia, cardiac ischemia. Meningitis, pancreatitis and rhabdomyolysis where removed from the model because they were recorded in less than 1% of control patients. Ethnicity was removed because it was not a good predictor in the model.

For peer review only

Appendix Table 6. Kaplan-Meier estimates of survival probability of hospitalised COVID-19 patients with and without an intellectual disability diagnosis

Time in hospital (days)	Survival probability (% and 95% CI)	
	Controls (n = 1484)	ID group (n = 472)
0	99.6 (99.3 - 99.9)	98.7 (97.7 - 99.7)
5	93.5 (92.1 - 95.0)	83.4 (79.9 - 87.0)
10	83.0 (80.5 - 85.6)	70.9 (66.5 - 75.7)
15	73.6 (70.3 - 77.0)	65.6 (60.7 - 70.8)
20	67.3 (63.5 - 71.3)	60.7 (55.4 - 66.6)

Appendix Table 7. Factors associated with hospital length of stay in COVID-19 patients

	**exp(β)**	**95% CI**	**p value**
Age group			
20-29 years old	1.23	1.10 - 1.37	**0.0002**
30-39 years old	1.30	1.17 - 1.43	**<0.0001**
40-49 years old	1.36	1.23 - 1.50	**<0.0001**
50-59 years old	1.40	1.28 - 1.54	**<0.0001**
60-69 years old	1.46	1.33 - 1.61	**<0.0001**
70-79 years old	1.48	1.34 - 1.65	**<0.0001**
80+ years old	1.69	1.49 - 1.92	**<0.0001**
Male sex	1.03	0.98 - 1.07	0.240
Shortness of breath	0.96	0.91 - 1.01	0.107
Respiratory rate	1.01	1.00 - 1.01	**0.0003**
No oxygen therapy	0.91	0.86 - 0.95	**<0.0001**
Number of comorbidities	1.05	1.04 - 1.07	**<0.0001**
DS diagnosis	1.08	0.95 - 1.22	0.229
ID diagnosis	1.15	1.09 - 1.22	**<0.0001**

For peer review only

Appendix Figure 2. Violin plot of the distribution of length of stay in COVID-19 patients with and without an intellectual disability diagnosis who were discharged alive

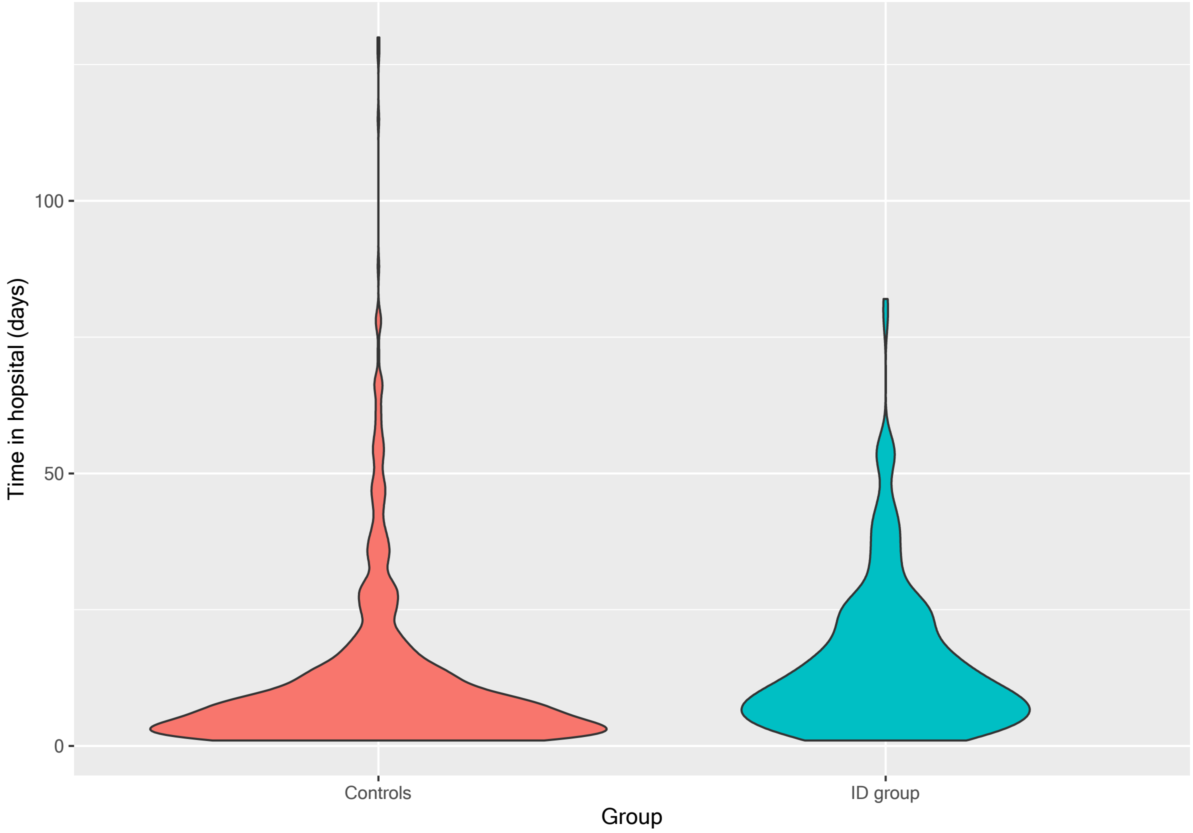

view only

STROBE Statement—Checklist of items that should be included in reports of ***cohort studies***

	Item No	Recommendation	Page No
**Title and abstract**	1	(*a*) Indicate the study's design with a commonly used term in the title or the abstract	1
		(*b*) Provide in the abstract an informative and balanced summary of what was done and what was found	2
**Introduction**			
Background/rationale	2	Explain the scientific background and rationale for the investigation being reported	3
Objectives	3	State specific objectives, including any prespecified hypotheses	3/4
**Methods**			
Study design	4	Present key elements of study design early in the paper	4
Setting	5	Describe the setting, locations, and relevant dates, including periods of recruitment, exposure, follow-up, and data collection	4
Participants	6	(*a*) Give the eligibility criteria, and the sources and methods of selection of participants. Describe methods of follow-up	4
		(*b*) For matched studies, give matching criteria and number of exposed and unexposed	
Variables	7	Clearly define all outcomes, exposures, predictors, potential confounders, and effect modifiers. Give diagnostic criteria, if applicable	4/5
Data sources/ measurement	8*	For each variable of interest, give sources of data and details of methods of assessment (measurement). Describe comparability of assessment methods if there is more than one group	4
Bias	9	Describe any efforts to address potential sources of bias	
Study size	10	Explain how the study size was arrived at	
Quantitative variables	11	Explain how quantitative variables were handled in the analyses. If applicable, describe which groupings were chosen and why	4
Statistical methods	12	(*a*) Describe all statistical methods, including those used to control for confounding	4
		(*b*) Describe any methods used to examine subgroups and interactions	
		(*c*) Explain how missing data were addressed	
		(*d*) If applicable, explain how loss to follow-up was addressed	
		(*e*) Describe any sensitivity analyses	
**Results**			
Participants	13*	(a) Report numbers of individuals at each stage of study—eg numbers potentially eligible, examined for eligibility, confirmed eligible, included in the study, completing follow-up, and analysed	
		(b) Give reasons for non-participation at each stage	
		(c) Consider use of a flow diagram	
Descriptive data	14*	(a) Give characteristics of study participants (eg demographic, clinical, social) and information on exposures and potential confounders	5
		(b) Indicate number of participants with missing data for each variable of interest	
		(c) Summarise follow-up time (eg, average and total amount)	
Outcome data	15*	Report numbers of outcome events or summary measures over time	

For peer review only - http://bmjopen.bmj.com/site/about/guidelines.xhtml

Main results	16	(*a*) Give unadjusted estimates and, if applicable, confounder-adjusted estimates and their precision (eg, 95% confidence interval). Make clear which confounders were adjusted for and why they were included<br>(*b*) Report category boundaries when continuous variables were categorized<br>(*c*) If relevant, consider translating estimates of relative risk into absolute risk for a meaningful time period	6-11
Other analyses	17	Report other analyses done—eg analyses of subgroups and interactions, and sensitivity analyses	
**Discussion**			
Key results	18	Summarise key results with reference to study objectives	11
Limitations	19	Discuss limitations of the study, taking into account sources of potential bias or imprecision. Discuss both direction and magnitude of any potential bias	13
Interpretation	20	Give a cautious overall interpretation of results considering objectives, limitations, multiplicity of analyses, results from similar studies, and other relevant evidence	14
Generalisability	21	Discuss the generalisability (external validity) of the study results	
**Other information**			
Funding	22	Give the source of funding and the role of the funders for the present study and, if applicable, for the original study on which the present article is based	14/5

*Give information separately for exposed and unexposed groups.

Note: An Explanation and Elaboration article discusses each checklist item and gives methodological background and published examples of transparent reporting. The STROBE checklist is best used in conjunction with this article (freely available on the Web sites of PLoS Medicine at http://www.plosmedicine.org/, Annals of Internal Medicine at http://www.annals.org/, and Epidemiology at http://www.epidem.com/). Information on the STROBE Initiative is available at http://www.strobe-statement.org.

For peer review only

For peer review only - http://bmjopen.bmj.com/site/about/guidelines.xhtml

BMJ Open

Understanding inequalities in COVID-19 outcomes following hospital admission for people with Intellectual disability compared to the general population: A matched cohort study in the United Kingdom

Journal:	*BMJ Open*
Manuscript ID	bmjopen-2021-052482.R1
Article Type:	Original research
Date Submitted by the Author:	08-Jul-2021
Complete List of Authors:	Baksh, R. Asaad; Institute of Psychiatry, Psychology, and Neuroscience, Department of Forensic and Neurodevelopmental Sciences, King's College London,<br>Pape, Sarah ; King's College London; South London and Maudsley NHS Foundation Trust<br>Smith, James; South London and Maudsley NHS Foundation Trust<br>Strydom, André; Institute of Psychiatry Psychology and Neuroscience
**Primary Subject Heading**:	Global health
Secondary Subject Heading:	Anaesthesia
Keywords:	INTENSIVE & CRITICAL CARE, Public health < INFECTIOUS DISEASES, PUBLIC HEALTH

SCHOLARONE™
Manuscripts

I, the Submitting Author has the right to grant and does grant on behalf of all authors of the Work (as defined in the below author licence), an exclusive licence and/or a non-exclusive licence for contributions from authors who are: i) UK Crown employees; ii) where BMJ has agreed a CC-BY licence shall apply, and/or iii) in accordance with the terms applicable for US Federal Government officers or employees acting as part of their official duties; on a worldwide, perpetual, irrevocable, royalty-free basis to BMJ Publishing Group Ltd ("BMJ") its licensees and where the relevant Journal is co-owned by BMJ to the co-owners of the Journal, to publish the Work in this journal and any other BMJ products and to exploit all rights, as set out in our licence.

The Submitting Author accepts and understands that any supply made under these terms is made by BMJ to the Submitting Author unless you are acting as an employee on behalf of your employer or a postgraduate student of an affiliated institution which is paying any applicable article publishing charge ("APC") for Open Access articles. Where the Submitting Author wishes to make the Work available on an Open Access basis (and intends to pay the relevant APC), the terms of reuse of such Open Access shall be governed by a Creative Commons licence – details of these licences and which Creative Commons licence will apply to this Work are set out in our licence referred to above.

Other than as permitted in any relevant BMJ Author's Self Archiving Policies, I confirm this Work has not been accepted for publication elsewhere, is not being considered for publication elsewhere and does not duplicate material already published. I confirm all authors consent to publication of this Work and authorise the granting of this licence.

For peer review only

Understanding inequalities in COVID-19 outcomes following hospital admission for people with Intellectual disability compared to the general population: A matched cohort study in the United Kingdom

R. Asaad Baksh,[1,3] Sarah E. Pape [1,2,3], James Smith,[2] & Andre Strydom [1,2,3]

[1.] *Institute of Psychiatry, Psychology, and Neuroscience, King's College London, London, UK.*

[2.] *South London and Maudsley NHS Foundation Trust, London, UK.*

[3.] *The LonDowns Consortium, London, UK.*

For peer review only

Corresponding author: Dr R. Asaad Baksh
Department of Forensic and Neurodevelopmental Sciences, Institute of Psychiatry, Psychology & Neuroscience, King's College London, London, SE5 8AF, UK
Email: asaad.baksh@kcl.ac.uk

Manuscript text: 4000 words (excluding, abstract, figures and tables)

For peer review only - http://bmjopen.bmj.com/site/about/guidelines.xhtml

ABSTRACT

OBJECTIVES: This study explores the hospital journey of patients with Intellectual disabilities (ID) compared to the general population after admission for COVID-19 during the first wave of the pandemic (when demand on inpatient resources was high) to identify disparities in treatment and outcomes.

DESIGN: Matched cohort study; an ID cohort of 506 patients were matched based on age, sex and ethnicity with a control group using a 1:3 ratio to compare outcomes from the International Severe Acute Respiratory and emerging Infections Consortium (ISARIC) World Health Organization (WHO) Clinical Characterisation Protocol UK (CCP-UK).

SETTING: Admissions for COVID-19 from UK hospitals; data on symptoms, severity, access to interventions, complications, mortality and length of stay were extracted.

INTERVENTIONS: Non-invasive respiratory support, intubation, tracheostomy, ventilation and admission to intensive care units (ICU).

RESULTS: Subjective presenting symptoms such as loss of taste/smell were less frequently reported in ID patients, whereas indicators of more severe disease such as altered consciousness and seizures were more common. Controls had higher rates of cardiovascular risk factors, asthma, rheumatologic disorder and smoking. ID patients were admitted with higher respiratory rates (Median=22, range=10–48) and were more likely to require oxygen therapy (35.1% vs 28.9%). Despite this, ID patients were 37% (13%–57% 95% CI) less likely to receive non-invasive respiratory support, 40% (7%–63% 95% CI) less likely to receive intubation and 50% (30%–66% 95% CI) less likely to be admitted to the ICU while in hospital. They had a 56% (17%–102% 95% CI) increased risk of dying from COVID-19 after they were hospitalised and were dying 1.44 times faster (1.13–1.84 95% CI) compared to controls.

CONCLUSIONS: There have been significant disparities in healthcare between people with ID and the general population during the COVID-19 pandemic, which may have contributed to excess mortality in this group.

For peer review only

ARTICLE SUMMARY

STRENGTHS AND LIMITATIONS OF THIS STUDY

- This is the first in-depth analysis of the hospital journey of patients with Intellectual disabilities compared to the general population after admission for COVID-19.
- We had a large sample size of 506 patients with intellectual disabilities and 1518 well-matched controls.
- Our dataset included data on comorbidities, vital signs, COVID-19 related admission signs and symptoms, complications due to COVID-19, information regarding interventions and outcome of hospitalisation.
- Due to data being collected at the time of care there was some degree of missing or incomplete data.

For peer review only

INTRODUCTION

Intellectually disability (ID) is a condition characterised by varying degrees of impairments in cognition, language, motor and social abilities depending on the severity of ID [1] and affects around 1% of the population globally [2]. Poorer health outcomes compared to the general population have been consistently reported for people with ID [3], with an increased incidence of comorbidities including dysphagia and respiratory diseases, with respiratory disease identified as a leading cause of death [4]. These health comorbidities are associated with poor outcomes following infections and other acute conditions [5 6], which may be exacerbated by barriers in accessing health and social care, associated with concerns about ongoing discrimination and bias [7].

To date there have been over 64 million cases of COVID-19 reported worldwide and 1.4 million deaths [8]. Several risk factors for increased mortality have been identified and reported [9], including increasing age [10], cardiovascular disease, chronic lung disease [11], cancer [12], chronic kidney disease [13] and obesity [14]. Evidence is now emerging that people with ID are disproportionally negatively impacted by COVID-19 [6 15 16]. The number of deaths of people with ID in England was three times higher in 2020 when compared to the corresponding period two years before [17] and people with ID may be more seriously affected by COVID-19 at a younger age than the general population [15 18]. Those with Down syndrome may be at particular risk of a more severe disease course, [19-21] specifically those 40 years and older [22]. Recent research has also suggested that people with Down syndrome have an increased risk of COVID-19 hospitalisation and death [23].

Given the existing health inequalities for people with ID, it is reasonable to further examine how people with COVID-19 and ID present to and progress through the acute hospital system and how this compares to the experiences of the general population. To date, only a few small-scale studies have examined the clinical presentation of COVID-19 in people with ID [15 16] and none have provided a comprehensive picture of their experiences once admitted to hospital for COVID-19. Specifically, there is little evidence relating to resources and treatment allocation for people with ID and how this compares to the general population.

Decisions around escalation of care, for example to Intensive Care Units (ICU), are complicated during a pandemic with added pressures related to rationing of resources. Such decisions have come under increasing scrutiny during the COVID-19 [24 25]. In the UK the NHS offered guidance to hospital trusts related to resource allocation [26], however there is little research about the impact of these guidelines on vulnerable populations such as people with ID.

The aim of our study was to explore the hospital journey of patients with ID compared to the general population after they were admitted to hospital for COVID-19 during the first wave of the pandemic, when pressure on health care systems were most acute. We have chosen to focus on interventions that require triaging and resource allocation, for both clinical and supply reasons [26-28]: non-invasive ventilation (NIV), tracheal intubation and admission to ICU. Comparisons were made to the general population in the following areas: 1) pattern and severity of COVID-19 symptoms at time of hospital admission; 2) comorbidities; 3) admission to intensive care and use of intubation and/or ventilation treatments; 4) complications during hospital admission; 5) outcomes of admission including length of stay and mortality.

Method

For peer review only - http://bmjopen.bmj.com/site/about/guidelines.xhtml

Study design and Setting

This study used data from The International Severe Acute Respiratory and emerging Infections Consortium (ISARIC) World Health Organization (WHO) Clinical Characterisation Protocol UK (CCP-UK). The ISARIC4C CCP-UK is an ongoing prospective cohort study in 260 hospitals across England, Scotland, and Wales (National Institute for Health Research Clinical Research Network Central Portfolio Management System ID 14152) [9]. The ISARIC4C CCP-UK protocol was activated on 17 January 2020 and information regarding the protocol, supplementary documents and details of the Independent Data and Material Access Committee (IDAMAC) are available at https://isaric4c.net.

Participants

The inclusion criteria for enrolment into the ISARIC4C CCP-UK cohort was patients of any age who were admitted to acute care hospitals with a proven or high likelihood of SARS-CoV-2 infection. Patients were admitted to hospital at the discretion of their clinical team and the study authors did not set criteria for inclusion. Patients who were already admitted to hospital for a separate clinical reason but had subsequently tested positive for COVID-19 during their stay were also included in the present study [9].

Overall in our sample were a total of 59,025 patients who were admitted between February 2020 and July 9, 2020 (downloaded on July 24, 2020). We identified 566 (0.96%) patients who had a diagnosis of ID and matched these patients to general population controls in the same dataset by age group, sex and ethnicity using a 1:3 ratio of ID patients to controls with no duplication of controls. Of the 566 ID patients, 506 had complete data on age group, sex and ethnicity and were matched to 1518 general population controls.

Data collection

Data were collected using a paper case report form that was developed by ISARIC4C CCP-UK and the WHO for use in outbreak investigations and uploaded to a REDCap database (Research Electronic Data Capture, Vanderbilt University, US, hosted by University of Oxford, UK). Consent from patients was not required to collect anonymised demographic and clinical data for research in England and Wales. For patients in Scotland, a waiver for consent was given by the Public Benefit and Privacy Panel.

Variables and data sources

Our dataset consisted of patient demographic information, comorbidities, vital signs, COVID-19 related admission signs and symptoms, complications due to COVID-19, information regarding interventions and outcome of hospitalisation. Data on these variables of interest were collected from the case report form developed by ISARIC4C CCP-UK and the WHO.

Patient and public involvement

The ISARIC4C CCP-UK study was as an urgent public health research study in response to a Public Health Emergency of International Concern, therefore patients were not involved in the design, conduct, or reporting of this rapid response research.

Bias and missing data

For peer review only

Criteria for the research team to enrol patients was based on local COVID-19 test reports, therefore the efficiency of testing labs may have biased patient enrolment. Data collection may have been limited by staff resourses at times of high COVID-19 infections. Due to the timing and nature of the study, there were missing or incomplete data, particularly as infection rates grew exponentially in the UK. Missing data was not imputed in the present study and consequently complete data were not available for all variables.

Data access and linkage

The study authors did not have direct access to the database population used to create the study population. Access to the study population data was granted by the Independent Data and Material Access Committee (https:// isaric4c.net). We did not conduct any data linkage for the present study.

Statistical Analysis

Descriptive statistics were used to show patient information, comorbidities and COVID-19 admission information, medical complications, interventions and outcomes. Statistical testing was performed using fisher's exact test for frequency data while Mann Whitney U was used for respiratory rate on admission and linear regression for frailty scores adjusted for age group and sex.

We conducted logistic regression modelling to examine whether demographic variables (age group and sex), severity of COVID-19 illness on admission (respiratory rate, need for oxygen therapy and shortness of breath), the number of comorbidities patients had on admission, a diagnosis of Down syndrome or an ID diagnosis were associated with COVID-19 related interventions. Similar logistic regression models were used to examine factors associated with mortality between groups, and with medical complications due to COVID-19. In the mortality between groups model we adjusted for significant mortality related comorbidities for COVID-19 that have been previously reported in the ISARIC4C CCP-UK dataset; these included chronic cardiac disease, chronic pulmonary disease, chronic kidney disease, liver disease, obesity, chronic neurological disorder, dementia and malignant neoplasm [9]. We reported risk ratios (RRs) with 95% confidence intervals (CIs) for the logistic regression models. Time-to-event analysis using Cox proportional hazards modelling was used to examine how soon after admission patients with ID were dying from COVID-19 compared to controls while adjusting for covariates (age group, sex, severity of COVID-19 on admission, number of comorbidities and DS diagnosis). We used death as the outcome and data were depicted with a Kaplan-Meier curve. Finally, potential differences in length of stay between ID patients and controls were explored using linear regression adjusting for the same covariates as the Cox proportional hazards model. To avoid violation of normality, clinical frailty scores and days in hospital was log-transformed and back transformed for reporting. All data analyses were done using R version 4.0.3.

Results

Description of study population and comorbidities

The sample of 506 ID patients consisted predominantly of adults over the age of 40 with only 25% of patients being under 40. Moreover, ID patients were mostly male and white, had lower rates of chronic cardiac disease, hypertension, chronic pulmonary disease, asthma, malignant

For peer review only - http://bmjopen.bmj.com/site/about/guidelines.xhtml

neoplasm, and rheumatologic disorders, and were less likely to be smokers than the general population controls (Table 1). On the other hand, higher rates of chronic neurological disorders (a broad category including cerebral palsy, multiple sclerosis, motor neurone disease, muscular dystrophy, myasthenia gravis, Parkinson's disease, stroke, severe learning difficulty) were reported in ID patients compared to controls, with a higher prevalence of dementia. The increased dementia rate is likely secondary to the association between Down syndrome and Alzheimer's disease included in the ID group.

For peer review only

		Controls		ID group		p value of comparison
		n	*N*	*n*	*N*	
		1518		506		
Age group (%)						
	<20	117 (7.7)		39 (7.7)		
	20-29	114 (7.5)		38 (7.5)		
	30-39	150 (9.9)		50 (9.9)		
	40-49	159 (10.5)		53 (10.5)		
	50-59	336 (22.1)		112 (22.1)		
	60-69	324 (21.3)		108 (21.3)		
	70-79	207 (13.6)		69 (13.6)		
	80+	111 (7.3)		37 (7.3)		
Sex (%)						
	Female	660 (43.5)		220 (43.5)		
	Male	858 (56.5)		286 (56.5)		
Ethnicity (%)						
	Aboriginal/First Nations	3 (0.2)		1 (0.2)		
	Black	36 (2.4)		12 (2.4)		
	East Asian	3 (0.2)		1 (0.2)		
	Other	96 (6.3)		32 (6.3)		
	South Asian	57 (3.8)		19 (3.8)		
	West Asian	9 (0.6)		3 (0.6)		
	White	1314 (86.6)		438 (86.6)		
Chronic cardiac disease		309 (21.5)	1439	81 (16.9)	479	**0.036**
Hypertension (physician diagnosed)		252 (31.9)	791	56 (18.7)	300	**<0.001**
Chronic pulmonary disease (not asthma)		191 (13.4)	1430	44 (9.2)	478	**0.016**

For peer review only - http://bmjopen.bmj.com/site/about/guidelines.xhtml

Asthma (physician diagnosed)	270 (18.8)	1435	68 (14.1)	481	**0.022**
Chronic kidney disease	140 (9.8)	1433	53 (11.0)	481	0.432
Mild, Moderate or severe liver disease[a]	54 (3.8)	1429	15 (3.1)	480	0.574
Diabetes[b]	266 (18.9)	1407	85 (18.2)	467	0.784
Chronic neurological disorder	156 (10.9)	1432	177 (36.6)	483	**<0.001**
Malignant neoplasm	100 (7.0)	1426	20 (4.2)	476	**0.029**
Chronic hematologic disease	39 (2.7)	1427	13 (2.7)	476	1.000
Obesity (as defined by clinical staff)	207 (15.7)	1317	69 (16.0)	431	0.879
Rheumatologic disorder	99 (6.9)	1426	20 (4.2)	473	**0.037**
Dementia	85 (5.9)	1437	47 (9.9)	473	**0.005**
Malnutrition	30 (2.2)	1378	12 (2.6)	459	0.590
Smoking					
Former Smoker	279 (26.4)	1055	43 (13.7)	313	**<0.001**
Never Smoked	676 (64.1)		247 (78.9)		
Yes	100 (9.5)		23 (7.3)		

The sample of 506 patients with an intellectual disability diagnosis from the UK ISARIC-4C matched to 1518 controls without an intellectual disability diagnosis based on age group, sex and ethnicity. [a]MiId, moderate and severe liver disease were combined into one category. [b]The variables diabetes and type, diabetes with complications, and diabetes without complications were combined into one category. The number of patients in the ID group with the comorbidities listed above on admission to hospital were compared to controls using Fisher's exact test.

Table 1. Characteristics of patients hospitalised for COVID-19 with and without an ID diagnosis

For peer review only

Signs, symptoms and severity of illness on admission in hospitalised COVID-19 patients with and without an ID diagnosis

A number of significant differences were observed in the symptoms at initial presentation to hospital between ID and control groups (Table 2). In particular, subjectively reported signs and symptoms such as loss of taste/smell, as well as those related to pain (headache, chest pain and muscle aches) were all reported less frequently in patients with ID. On the other hand, altered consciousness or confusion (29.9% vs 17.6%) and seizures (9.9% vs 2.2%) were more common in patients with ID. Compared to controls, ID patients were admitted with higher respiratory rates and were more likely to require oxygen therapy. In addition, adjusted for age group and sex, having a diagnosis of ID was significantly associated with higher clinical frailty scores.

	Controls		ID group		p value of comparison
	n (%)	N	n (%)	N	
Cough	972 (67.6)	1438	309 (64.6)	478	0.239
Cough with sputum production*	285 (22.7)	1254	58 (14.6)	397	**<0.001**
Cough with bloody sputum	41 (3.3)	1240	9 (2.3)	393	0.401
Fever	1004 (69.6)	1442	335 (69.8)	480	1.000
Sore throat	123 (10.4)	1186	29 (8.0)	364	0.191
Runny nose*	49 (4.2)	1168	6 (1.7)	357	**0.023**
Wheezing	94 (7.7)	1228	41 (10.1)	407	0.145
Ear pain	7 (0.6)	1150	3 (0.8)	364	0.711
Chest pain*	225 (17.8)	1267	35 (8.7)	404	**<0.001**
Muscle aches*	275 (23.1)	1192	30 (8.4)	357	**<0.001**
Joint pain	70 (6.1)	1147	18 (5.1)	356	0.520
Fatigue	511 (40.7)	1254	145 (37.5)	387	0.260
Shortness of breath*	953 (67.3)	1416	274 (59.8)	458	**0.004**
Disturbance or loss of taste*	51 (8.8)	578	3 (1.4)	207	**<0.001**
Disturbance or loss of smell*	36 (6.1)	588	1 (0.5)	212	**<0.001**
Headache*	177 (14.9)	1184	20 (5.5)	362	**<0.001**
Altered consciousness or confusion*	233 (17.6)	1326	124 (29.9)	415	**<0.001**
Seizures	28 (2.2)	1291	41 (9.9)	415	**<0.001**
Abdominal pain	187 (14.6)	1280	53 (13.2)	403	0.514
Vomiting and/or Nausea*	323 (24.3)	1329	67 (15.7)	426	**<0.001**
Diarrhoea*	279 (21.0)	1327	58 (13.4)	432	**<0.001**
Conjunctivitis	11 (0.9)	1205	4 (1.0)	384	0.767
Lymphadenopathy	10 (0.8)	1206	0 (0.0)	390	0.131
Skin rash	33 (2.7)	1228	8 (2.0)	396	0.581
Skin ulcers	19 (1.5)	1231	6 (1.5)	401	1.000
Haemorrhage	19 (1.5)	1261	4 (1.0)	416	0.626
Requirement of oxygen therapy on admission	406 (28.9)	1407	170 (35.1)	484	**0.011**
Median respiratory rate (breaths per minute) on admission (interquartile range)[a]	21 (10-50)	1404	22 (10-48)	464	**0.009**

For peer review only - http://bmjopen.bmj.com/site/about/guidelines.xhtml

Mean clinical frailty score (SD)	3.55 (2.17)	437	5.14 (1.89)	175	**<0.0001**

The number of patients in the ID group presenting with COVID-19 related symptoms on admission to hospital, compared to controls using Fisher's exact test. [a]We excluded respiratory rate values that were below 10 or higher than 50 breaths per minute as such data were considered outliers.

Table 2. Admission signs, symptoms and severity of illness on admission related to COVID-19 in hospitalised patients with and without an ID diagnosis

Medical complications among hospitalised COVID-19 patients with and without an ID diagnosis

In both the ID and general population groups the leading complications due to COVID-19 (Appendix Table 1) were pulmonary complications including viral pneumonia, bacterial pneumonia and acute respiratory syndrome, as well as acute renal injury and/or acute renal failure, anaemia and cardiac complications. Overall, medical complications were comparable between patients with ID and controls, with the exception of seizures which were more prevalent in the ID group (5.1% of those with ID compared to 2.0% of the control group).

Factors associated with COVID-19 related interventions

An increased likelihood of admission to ICU, tracheal intubation and non-invasive respiratory support were all associated with higher respiratory rate, shortness of breath and the requirement of oxygen therapy on admission, suggesting that the severity of illness on admission is important for prognosis and the need for COVID-19 related interventions. Significantly fewer ID patients were admitted to ICU, underwent intubation or received non-invasive respiratory support compared to controls (Table 3). Adjusted for age group, sex, severity of illness on admission, number of comorbidities and DS diagnosis, patients with ID were 37% less likely to receive non-invasive respiratory support, 40% less likely to receive intubation and 50% less likely to be admitted to the ICU while in hospital (Figure 1).

	**Controls**		**ID group**		**p value of**
	**n**	**N**	**n**	**N**	**comparison**
Non-invasive respiratory support	243 (16.9)	1436	60 (12.3)	487	**0.017**
Tracheal intubation	167 (11.2)	1496	36 (7.2)	503	**0.010**
Tracheostomy	16 (2.5)	637	2 (1.1)	178	0.390
Any time in intensive care unit	304 (20.3)	1500	59 (11.7)	505	**<0.001**

Table 3. COVID-19 related interventions for hospitalised patients with and without an intellectual disability diagnosis

Mortality rates and factors associated with mortality among COVID-19 patients with and without an ID diagnosis

People with ID had a 56% increased risk of dying from COVID-19 after they were hospitalised compared to controls, with a mortality rate of 29.2% for the ID group compared to 18.8% for

For peer review only - http://bmjopen.bmj.com/site/about/guidelines.xhtml

controls (Appendix Figure 1.). Adjusted for age group, sex, known mortality related comorbidities, severity of illness on admission, interventions and DS diagnosis, the association between mortality and an ID diagnosis remained significant (Appendix Table 2).

Examining the factors associated with mortality in the ID group only we found that age (50 years and older), requiring oxygen therapy and higher respiratory rates at admission were all significantly associated with increased risk of dying from COVID-19. None of the known mortality-related comorbidities were significantly associated with mortality in patients with ID in our sample (Appendix Table 3).

Insert Figure 1 around here

Associations between medical complications and mortality

Viral pneumonia was significantly associated with mortality in the ID group. This complication increased ID patients' risk of dying by 174%. Acute respiratory syndrome was also strongly associated with mortality and increased ID patients' risk of dying by 107% (Appendix Table 4).

In comparison, while still significantly associated with mortality in controls, viral pneumonia was associated with a 56% increase in risk of dying and acute respiratory syndrome increased risk of dying by 91%. On the other hand, cardiac arrest was associated with a 438% increase risk of dying in controls, gastrointestinal haemorrhage increased the risk of dying by 178%, acute renal injury by 99% and other cardiac complications by 82% (Appendix Table 5).

Survival analysis of COVID -19 patients with and without an ID diagnosis

After five days in hospital, 16.6% of ID patients had died compared to only 6.5% of controls. This trend continued so that at twenty days 39.3% of ID patients had died compared to 32.7% of controls (Appendix Table 6). Figure 2 shows the Kaplan-Meier estimates of survival probability for our ID group and controls. Adjusting for age group, sex, DS diagnosis, number of comorbidities and severity of COVID-19 on admission, the hazard ratio (HR) for COVID-19 related mortality in patients with ID compared to controls was 1.44 (95% CI = 1.13 - 1.84, p = 0.003**).** Therefore, patients with ID were dying 1.44 times faster than controls at any particular point in time after they were admitted to hospital for COVID-19, even after adjusting for covariates.

Insert Figure 2 around here

Factors associated with length of time in hospital for COVID-19 patients with and without an ID diagnosis

A significant association between a diagnosis of ID and length of time in hospital was found, with ID patients spending longer periods in hospital after they were admitted for COVID-19 (Table 4). The controls spent a mean of 10.98 days in hospital (SD = 14.45, median = 6.5 days) while the ID group spent 14.55 days on average (SD = 13.29, median = 11 days; Appendix Figure 2). Other factors significantly associated with longer stays in hospital in both groups were being older than 20 years, more comorbidities and greater severity of illness on admission.

For peer review only - http://bmjopen.bmj.com/site/about/guidelines.xhtml

	exp(β)	95% CI	p value
Age group			
20-29 years old	1.23	1.10 - 1.37	**0.0002**
30-39 years old	1.30	1.17 - 1.43	**<0.0001**
40-49 years old	1.36	1.23 - 1.50	**<0.0001**
50-59 years old	1.40	1.28 - 1.54	**<0.0001**
60-69 years old	1.46	1.33 - 1.61	**<0.0001**
70-79 years old	1.48	1.34 - 1.65	**<0.0001**
80+ years old	1.69	1.49 - 1.92	**<0.0001**
Male sex	1.03	0.98 - 1.07	0.240
Shortness of breath	0.96	0.91 - 1.01	0.107
Respiratory rate	1.01	1.00 - 1.01	**0.0003**
No oxygen therapy	0.91	0.86 - 0.95	**<0.0001**
Number of comorbidities	1.05	1.04 - 1.07	**<0.0001**
DS diagnosis	1.08	0.95 - 1.22	0.229
ID diagnosis	1.15	1.09 - 1.22	**<0.0001**

Table 4. Factors associated with hospital length of stay in COVID-19 patients

DISCUSSION

This is the first in-depth exploration of treatment and interventions offered to patients with ID who were admitted to hospital for COVID-19. We found that the hospital journey for people with ID and COVID-19 is substantially different to the general population in a number of fundamental areas: recognition and assessment of COVID-19 symptoms; symptoms and severity of illness on admission; access to interventions and ICUs; mortality rates, survival trajectories and duration of hospital stay.

Recognition and Assessment of COVID-19 Symptoms

The most prevalent symptoms recorded at admission in both the ID and control group were cough, fever and shortness of breath, in keeping with previous reports [29]. However, patients with ID were significantly less likely to present with subjective symptoms including pain, loss of taste or smell, and 'shortness of breath', despite having higher respiratory rates at admission. People with ID were more likely to present with altered consciousness, confusion and seizures which could indicate a more severe presentation upon admission. Patients with ID also presented with other indicators of more severe illness at the point of admission, including greater requirement for supplemental oxygen therapy and increased average respiratory rates compared to controls. This could represent late presentation to hospital by people with ID. There are several potential explanations for late presentation of patients with ID: poor symptom recognition by caregivers and patients themselves, communication difficulties, and exclusion from digital information and public health campaigns which could reduce awareness about early warning signs and symptoms. Other issues which may have contributed to later referral to hospital include a reluctance from family members to hospitalise their relative or disability discrimination resulting in people with ID not being able to access medical services.

Course of illness in hospitalised patients with ID and access to Interventions and Intensive Care Units

For peer review only - http://bmjopen.bmj.com/site/about/guidelines.xhtml

Once admitted, patients with ID and COVID-19 had a more aggressive course of disease, with higher rates of death in the early stages of hospitalisation as well as longer hospital stays. Rates of complications and most comorbidities were comparable between the groups, however patients with ID were given higher scores on the clinical frailty scale, potentially reflecting misinterpretation of the degree of frailty in the context of long-term but stable cognitive impairment. This has implications for treatment decisions around resource allocation when availability may be limited.

Despite having more severe symptoms upon admission and similar rates of complications, patients with ID were less likely to be treated with non-invasive ventilation, tracheal intubation, or be admitted to an ICU setting. This disparity in access to appropriate treatment has been highlighted in investigations of other conditions [30], with issues surrounding decision-making capacity, ceilings of care, inappropriate use of clinical frailty scales, and discrimination or biases potentially contributing to inequalities in care [31]. Other contributing factors may be related to tolerability of interventions (particularly NiV) for people with ID, perceived treatment difficulties that may influence decision making, and inappropriate use of Do Not Resuscitate orders (DNaCPRs) [32].

Complications of COVID-19 Infection, Mortality Rates, and Length of Stay

Having a diagnosis of ID was associated with a 56% increase in mortality risk, which was not associated with seizures or dementia, despite these conditions being more common in ID patients compared to the general population, particularly those with Down syndrome [33]. The increased mortality also does not appear to be related to other suggested COVID-19 comorbidities for adverse outcome [9 11 13], although as in the general population, older age and severity of illness on admission did show significant associations with mortality in ID. As well as an increased mortality rate in ID patients after admission to hospital, we found a different survival trajectory. ID patients died at a 1.44 times faster rate than the general population, even when age, comorbidities and severity of symptoms were considered. This suggests that aspects of their care and treatment may be contributing to increased mortality rather than co-morbidities or complications.

People with ID who survived had a longer inpatient stay on average. Again, this does not appear to be secondary to increased complications or co-morbidities. It is therefore possible that people with ID may be experiencing delays in their discharge and support to return to the community. Longer admissions can be associated with distress for the individual, exposure to risk of hospital acquired infections, and institutionalisation. These findings highlight the different experiences of patients with ID after they were admitted to hospital for COVID-19 compared to the general population.

Strengths and limitations

The strengths of the study are the large sample size and the use of a well-matched control group which allows for comparisons in symptoms, treatment and outcomes to be captured. Data was taken from across the UK meaning it is reflective of experiences across the country rather than regionally specific issues. It used real-world data captured during an acute and evolving pandemic and gives insight into conditions faced by patients and health professionals at the time.

For peer review only - http://bmjopen.bmj.com/site/about/guidelines.xhtml

Some limitations are acknowledged. The study relied upon data captured at the time of care. Whilst this provides an accurate picture of acute clinical care, the nature of clinical records can lead to some degree of missing or incomplete data. In addition, the use of combined group categories (particularly the heterogenous group "chronic neurological disorder") limited the ability to explore the potential impact of specific diseases, while the reason for specific clinical decisions may not be clear. Further research is therefore needed to explore the details around clinical decision making for people with ID during pandemic conditions and the impact of care rationalisation on this population. It will also be important to understand the experiences of individuals with ID and role and experience of their caregivers, particularly with regards to decision making, advocacy and inclusion. As ISARIC4C CCP-UK is a UK population-based study and not specifically focused on people with ID, we were unable to consider the extent to which issues particularly relevant to people with ID such as availability of different modes of care, supported decision-making or the presence of family members or other close supportive persons to help with isolation and understanding of the pandemic may have affected our results. Further work is needed to examine how these factors may impact those admitted to hospital for COVID-19.

Conclusion

These findings highlight an ongoing disparity in healthcare between people with ID and the general population which have been magnified by the COVID-19 pandemic, with implications for improving care and treatment during the ongoing crisis to ensure the levelling-up of services for the future. It is hard not to be concerned at the possibility of bias and discrimination affecting treatment decisions in such conditions, whether implicit or explicit. Barriers to care will need to be overcome and information should be disseminated in an accessible way to both caregivers and people with ID, particularly with regards to early symptoms and warning signs of a more severe presentation. In the community digital exclusion has been identified as a barrier to information for people with ID [34]. This may make it more difficult for people with ID to report early signs, receive up to date information about risks, or indeed even be part of track-and-trace systems. They may also be less able to self-monitor for early signs such as fevers. Moves towards the use of home oxygen saturation monitoring may be helpful in this population in identifying at risk people before they become acutely unwell and could allow for treatment to be initiated in a timely manner to reduce mortality.

Similarly, the results stress the need for people with ID admitted for COVID-19 (and other similar infections) to be prioritised for enhanced care and monitoring based on indicators of deterioration, without reliance on self-reporting. Earlier intervention may be indicated to avoid the more aggressive course of illness. Provisions and training should be in place in all hospitals regarding capacity and decision making, and trained staff should be available to assist in these matters. Echoing the recommendations of other researchers [35], people with ID should be prioritised for COVID-19 vaccinations and boosters in the future. Care should be taken when making decisions about prioritisation of interventions to ensure they are not biased against people with long-term disabilities, but instead based on relevant prognostic indicators. Medical ethics panels which include professionals who are familiar with the care and needs of people with ID could assist with such decisions.

It is hoped that these results from the first wave of the pandemic highlight the ongoing health disparities faced by people with ID and will help raise awareness and mobilise health care services to improve practices and access for this population.

For peer review only - http://bmjopen.bmj.com/site/about/guidelines.xhtml

Ethical approval

Ethical approval was given by the South Central - Oxford C Research Ethics Committee in England (ref 13/SC/0149), the Scotland A Research Ethics Committee (ref 20/SS/0028), and the WHO Ethics Review Committee (RPC571 and RPC572, 25 April 2013).

Data sharing

The Independent Data and Material Access Committee welcomes applications for access to data and materials (https:// isaric4c.net).

Competing interests

There are no competing interests for any author

Author Contributions

AS conceived and designed the project with help from RAB. RAB and AS planned the data analysis. RAB conducted the data analysis. RAB, SP and JS wrote the first draft with input from AS. All authors contributed to reviewing and revising the manuscript and agreed final approval of the version to be published.

Acknowledgements

This report is independent research which used data provided by the MRC funded ISARIC 4C Consortium and which the Consortium collected under a research contract funded by the National Institute for Health Research. The views expressed in this publication are those of the authors and not necessarily those of the ISARIC 4C consortium.

Funding

This study was supported by grants from the MRC (MR/S011277/1; MR/S005145/1; MR/R024901/1), the European Commission (H2020 SC1 Gene overdosage and comorbidities during the early lifetime in Down Syndrome GO-DS21- 848077) and Alzheimer's Society (AS-CP-18-0020: fellowship to SP).

This study represents independent research part funded by the National Institute for Health Research (NIHR) Maudsley Biomedical Research Centre at South London and Maudsley NHS Foundation Trust and King's College London. The views expressed are those of the authors and not necessarily those of the NHS, the NIHR or the Department of Health and Social Care.

REFERENCES

1. World Health Organization. The International Classification of Diseases – Tenth revision (ICD10). Geneva: World Health Organization 1992.

For peer review only - http://bmjopen.bmj.com/site/about/guidelines.xhtml

2. Maulik PK, Mascarenhas MN, Mathers CD, et al. Prevalence of intellectual disability: a meta-analysis of population-based studies. *Research in developmental disabilities* 2011;32(2):419-36.
3. Heslop P, Blair PS, Fleming P, et al. The Confidential Inquiry into premature deaths of people with intellectual disabilities in the UK: a population-based study. *The Lancet* 2014;383(9920):889-95.
4. Cooper S-A, Allan L, Greenlaw N, et al. Rates, causes, place and predictors of mortality in adults with intellectual disabilities with and without Down syndrome: cohort study with record linkage. *BMJ Open* 2020;10(5):e036465. doi: 10.1136/bmjopen-2019-036465
5. Cuypers M, Schalk B, Koks-Leensen M, et al. Mortality of people with intellectual disabilities during the 2017/2018 influenza epidemic in the Netherlands: potential implications for the COVID-19 pandemic. *Journal of Intellectual Disability Research* 2020;64(7):482-88.
6. Landes SD, Turk MA, Formica MK, et al. COVID-19 outcomes among people with intellectual and developmental disability living in residential group homes in New York State. *Disability and Health Journal* 2020;13(4):100969.
7. Amon JJ, Wurth M. A Virtual Roundtable on COVID-19 and Human Rights with Human Rights Watch Researchers. *Health Hum Rights* 2020;22(1):399-413.
8. Dong E, Du H, Gardner L. An interactive web-based dashboard to track COVID-19 in real time. *The Lancet Infectious Diseases* 2020;20(5):533-34. doi: https://doi.org/10.1016/S1473-3099(20)30120-1
9. Docherty AB, Harrison EM, Green CA, et al. Features of 20 133 UK patients in hospital with covid-19 using the ISARIC WHO Clinical Characterisation Protocol: prospective observational cohort study. *bmj* 2020;369
10. Verity R, Okell LC, Dorigatti I, et al. Estimates of the severity of coronavirus disease 2019: a model-based analysis. *The Lancet infectious diseases* 2020;20(6):669-77.
11. Yang J, Zheng Y, Gou X, et al. Prevalence of comorbidities and its effects in patients infected with SARS-CoV-2: a systematic review and meta-analysis. *International Journal of Infectious Diseases* 2020;94:91-95.
12. Fung M, Babik JM. COVID-19 in immunocompromised hosts: what we know so far. *Clinical Infectious Diseases* 2021;72(2):340-50.
13. Richardson S, Hirsch JS, Narasimhan M, et al. Presenting characteristics, comorbidities, and outcomes among 5700 patients hospitalized with COVID-19 in the New York City area. *Jama* 2020;323(20):2052-59.
14. Simonnet A, Chetboun M, Poissy J, et al. High prevalence of obesity in severe acute respiratory syndrome coronavirus-2 (SARS-CoV-2) requiring invasive mechanical ventilation. *Obesity* 2020;28(7):1195-99.
15. Perera B, Laugharne R, Henley W, et al. COVID-19 deaths in people with intellectual disability in the UK and Ireland: descriptive study. *BJPsych open* 2020;6(6)
16. Turk MA, Landes SD, Formica MK, et al. Intellectual and developmental disability and COVID-19 case-fatality trends: TriNetX analysis. *Disability and Health Journal* 2020;13(3):100942.
17. Public Health England. Deaths of people identified as having learning disability with COVID-19 in England in the spring of 2020. https://assets.publishing.service.gov.uk/government/uploads/system/uploads/attachment_data/file/933612/COVID-19__learning_disabilities_mortality_report.pdf 2020.
18. Care Quality Commission. CQC publishes data on deaths of people with a learning disability: Care Quality Commission; 2020 [Available from:

https://www.cqc.org.uk/news/stories/cqc-publishes-data-deaths-people-learning-disability accessed 24 March 2021 2021.
19. De Cauwer H, Spaepen A. Are patients with Down syndrome vulnerable to life-threatening COVID-19? *Acta Neurologica Belgica* 2020:1-3.
20. Illouz T, Biragyn A, Frenkel-Morgenstern M, et al. Specific Susceptibility to COVID-19 in Adults with Down Syndrome. *Neuromolecular medicine* 2021:1-11.
21. Kantar A, Mazza A, Bonanomi E, et al. COVID-19 and children with Down syndrome: is there any real reason to worry? Two case reports with severe course. *BMC pediatrics* 2020;20(1):1-5.
22. Hüls A, Costa AC, Dierssen M, et al. Medical vulnerability of individuals with Down syndrome to severe COVID-19–data from the Trisomy 21 Research Society and the UK ISARIC4C survey. *EClinicalMedicine* 2021;33:100769.
23. Clift AK, Coupland CA, Keogh RH, et al. COVID-19 mortality risk in Down syndrome: results from a cohort study of 8 million adults. *Annals of internal medicine* 2020
24. Griffiths F, Svantesson M, Bassford C, et al. Decision-making around admission to intensive care in the UK pre-COVID-19: a multicentre ethnographic study. *Anaesthesia* 2020;76(4) doi: 10.1111/anae.15272
25. Montgomery J, Stokes-Lampard H, Griffiths M, et al. Assessing whether COVID-19 patients will benefit from critical care, and an objective approach to capacity challenges during a pandemic: an intensive care Society clinical guideline. *Journal of the Intensive Care Society* 2020;0(0):1-7.
26. I.C.M. Anaesthesia. Clinical Guide for the Management of Critical Care for Adults with COVID-19 During the Coronavirus Pandemic. https://icmanaesthesiacovid-19.org/clinical-guide-for-the-management-of-critical-care-for-adults-with-covid-19-during-the-coronavirus-pandemic: ICM Anaesthesia Covid-19, 2020.
27. Phua J, Weng L, Ling L, et al. Intensive care management of coronavirus disease 2019 (COVID-19): challenges and recommendations. *The Lancet Respiratory Medicine* 2020;8(5):506-17.
28. White DB, Lo B. A framework for rationing ventilators and critical care beds during the COVID-19 pandemic. *Jama* 2020;323(18):1773-74.
29. Stokes EK, Zambrano LD, Anderson KN, et al. Coronavirus disease 2019 case surveillance—United States, January 22–May 30, 2020. *Morbidity and Mortality Weekly Report* 2020;69(24):759.
30. LeDeR. Deaths of people with learning disabilities from COVID-19. http://www.bristol.ac.uk/sps/leder/news/2020/leder-covid-19-reviews.html, 2020.
31. Ali A, Scior K, Ratti V, et al. Discrimination and other barriers to accessing health care: perspectives of patients with mild and moderate intellectual disability and their carers. *PloS one* 2013;8(8):e70855.
32. Chen B, McNamara DM. Disability discrimination, medical rationing and COVID-19. *Asian bioethics review* 2020;12(4):511-18.
33. Firth NC, Startin CM, Hithersay R, et al. Aging related cognitive changes associated with Alzheimer's disease in Down syndrome. *Annals of clinical and translational neurology* 2018;5(6):741-51.
34. Caton S, Chapman M. The use of social media and people with intellectual disability: A systematic review and thematic analysis. *Journal of Intellectual & Developmental Disability* 2016;41(2):125-39. doi: 10.3109/13668250.2016.1153052
35. De Cauwer H, Rodiers A, Spaepen A. Response to Covid-19: What is happening with the vaccine rollout? *BMJ* 2021;372:n213.

Figures title and caption:

For peer review only

Figure 1. Factors associated with interventions (non-invasive respiratory support, intubation and ICU) in hospitalised COVID-19 patients with and without an intellectual disability diagnosis

A: *Factors associated with access to non-invasive respiratory support (e.g. BIPAP, CPAP).* ***B:*** *Factors associated with the use of tracheal intubation.* ***C****: Factors associated with admission to ICU. Bold labels on the forest plots indicate statistically significant associations. Percent relative effects can be calculated using (RR - 1) x 100 for RRs over 1 or (1 - RR) x 100 for RRs less than 1. For example, shortness of breath on admission was associated with a 73% [(1.73-1) x 100] increase in risk of being admitted to the ICU while not requiring oxygen therapy of admission was associated with a 48% [(1-0.52) x 100] decrease in risk of being admitted to the ICU while in hospital. We present log-transformed RRs in the plots.*

Figure 2. Kaplan-Meier survival plot of hospitalised COVID-19 patients with and without an intellectual disability diagnosis

For peer review only

For peer review only - http://bmjopen.bmj.com/site/about/guidelines.xhtml

A

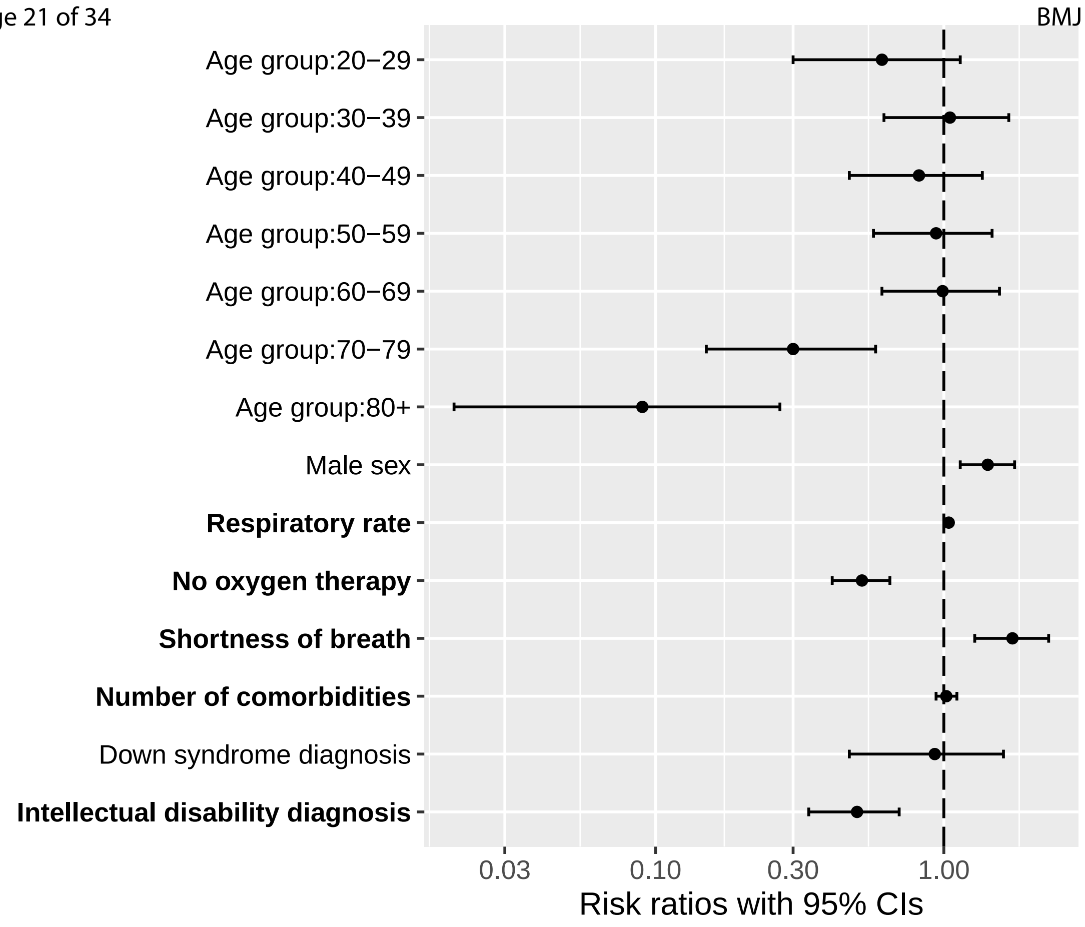

	Risk ratio	Lower CI	Upper CI	p value
Age group:20−29	1.13	0.48	2.47	0.771
Age group:30−39	1.31	0.63	2.62	0.474
Age group:40−49	1.33	0.65	2.63	0.435
Age group:50−59	1.73	0.93	3.18	0.095
Age group:60−69	1.79	0.96	3.27	0.079
Age group:70−79	0.83	0.39	1.77	0.630
Age group:80+	0.50	0.19	1.26	0.147
Male sex	1.27	1.00	1.59	0.053
Respiratory rate	1.04	1.02	1.06	<0.0001
No oxygen therapy	0.56	0.43	0.70	<0.0001
Shortness of breath	2.10	1.50	2.93	<0.0001
Number of comorbidities	1.14	1.05	1.24	0.002
Down syndrome diagnosis	1.38	0.76	2.22	0.261
Intellectual disability diagnosis	0.63	0.43	0.87	0.007

B

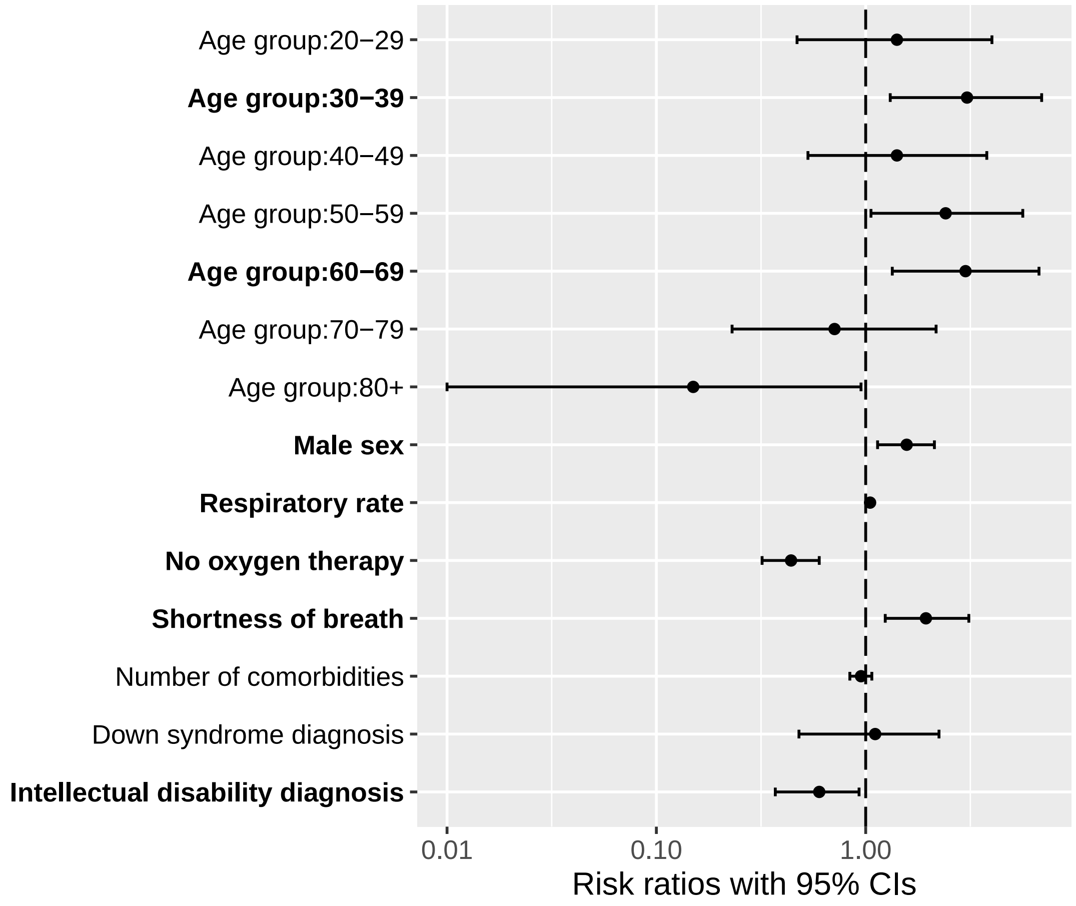

	Risk ratio	Lower CI	Upper CI	p value
Age group:20−29	1.41	0.47	4.01	0.536
Age group:30−39	3.05	1.31	6.93	0.015
Age group:40−49	1.41	0.53	3.79	0.499
Age group:50−59	2.41	1.06	5.63	0.051
Age group:60−69	3.00	1.34	6.73	0.014
Age group:70−79	0.71	0.23	2.17	0.538
Age group:80+	0.15	0.01	0.95	0.086
Male sex	1.57	1.14	2.13	0.006
Respiratory rate	1.05	1.03	1.07	<0.001
No oxygen therapy	0.44	0.32	0.60	<0.0001
Shortness of breath	1.94	1.24	3.11	0.006
Number of comorbidities	0.95	0.84	1.07	0.396
Down syndrome diagnosis	1.11	0.48	2.24	0.795
Intellectual disability diagnosis	0.60	0.37	0.93	0.028

C

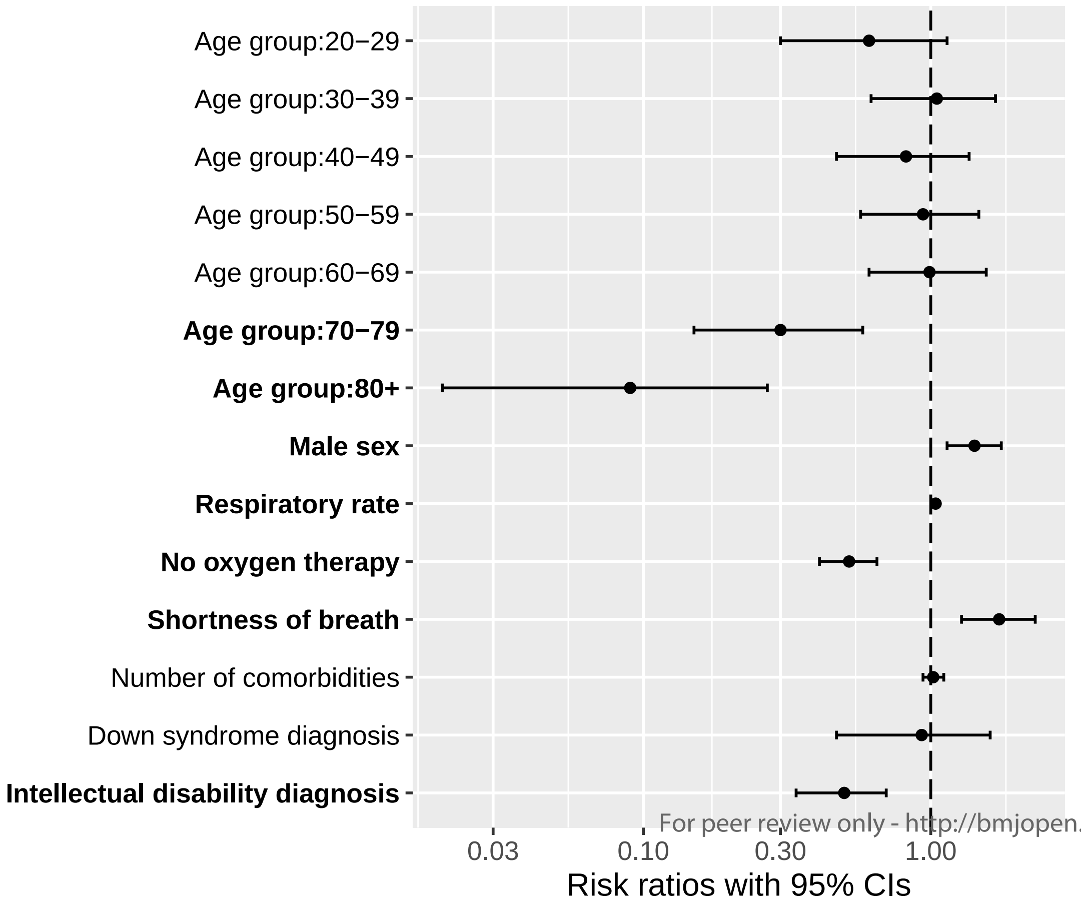

	Risk ratio	Lower CI	Upper CI	p value
Age group:20−29	0.61	0.30	1.14	0.131
Age group:30−39	1.05	0.62	1.68	0.852
Age group:40−49	0.82	0.47	1.36	0.454
Age group:50−59	0.94	0.57	1.47	0.786
Age group:60−69	0.99	0.61	1.56	0.982
Age group:70−79	0.30	0.15	0.58	0.0002
Age group:80+	0.09	0.02	0.27	<0.0001
Male sex	1.42	1.14	1.76	0.002
Respiratory rate	1.04	1.03	1.06	<0.0001
No oxygen therapy	0.52	0.41	0.65	<0.0001
Shortness of breath	1.73	1.28	2.31	0.0005
Number of comorbidities	1.02	0.94	1.11	0.584
Down syndrome diagnosis	0.93	0.47	1.61	0.810
Intellectual disability diagnosis	0.50	0.34	0.70	<0.0001

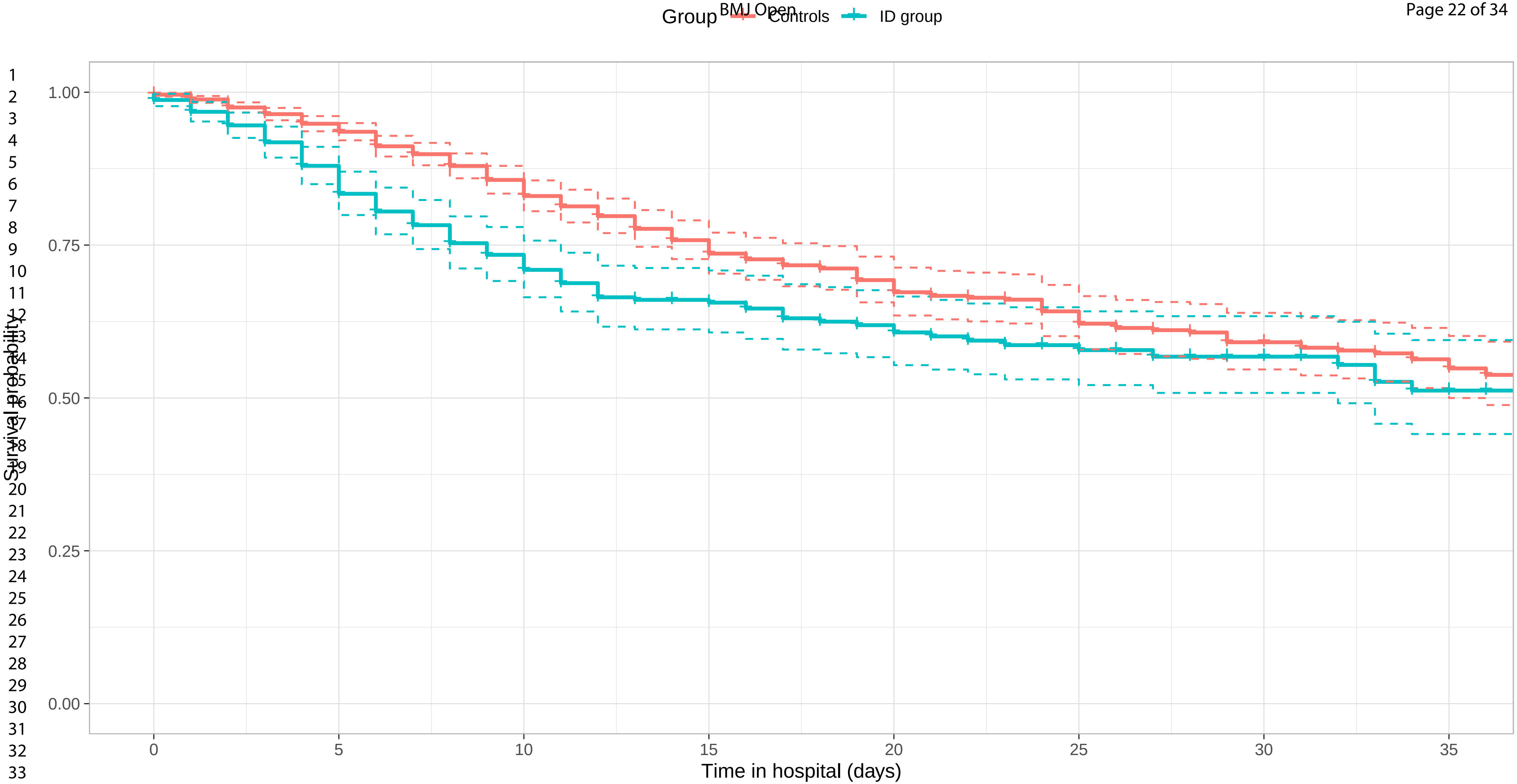

Number at risk: n (%)

Group	0	5	10	15	20	25	30	35
Controls	1484 (100)	939 (63)	552 (37)	344 (23)	244 (16)	191 (13)	141 (10)	113 (8)
ID group	472 (100)	346 (73)	209 (44)	144 (31)	104 (22)	72 (15)	47 (10)	35 (7)

Time in hospital (days)

Appendix Table 1. Complications related to COVID-19 in hospitalised patients with and without an ID diagnosis

	Controls		ID group		p value of comparison
	n (%)	N	n (%)	N	
Viral pneumonia	595 (43.3)	1375	222 (47.4)	468	0.119
Bacterial pneumonia	153 (11.2)	1364	63 (13.6)	463	0.182
Acute respiratory syndrome	160 (11.6)	1382	44 (9.3)	471	0.201
Other lung complications[1]	97 (6.9)	1408	28 (5.9)	476	0.523
Meningitis / Encephalitis	6 (0.4)	1396	1 (0.2)	474	0.687
Seizures	28 (2.0)	1401	24 (5.1)	474	**0.001**
Other neurological complications[2]	31 (2.2)	1401	7 (1.5)	472	0.450
Cardiac arrest	31 (2.2)	1397	9 (1.9)	473	0.854
Other cardiac complications[3]	132 (9.4)	1409	34 (7.2)	473	0.160
Bacteraemia	40 (2.9)	1391	10 (2.1)	469	0.509
Gastrointestinal hemorrhage or coagulation disorder	44 (3.1)	1402	16 (3.4)	473	0.764
Pancreatitis	10 (0.7)	1395	1 (0.2)	473	0.309
Rhabdomyolysis / Myositis	5 (0.4)	1395	2 (0.4)	473	1.000
Anaemia	164 (11.7)	1404	46 (9.7)	473	0.273
Acute renal injury and/or acute renal failure	192 (13.7)	1402	57 (12.0)	475	0.389
Liver dysfunction	60 (4.3)	1396	16 (3.4)	472	0.422
Hypoglycaemia or hyperglycaemia	88 (6.3)	1386	30 (6.3)	473	1.000

[1]Combined cryptogenic organizing pneumonia (COP), pneumothorax, pleural effusion and bronchiolitis, [2]Combined Stroke / Cerebrovascular accident and other neurological complication, [3]Combined congestive heart failure, endocarditis / myocarditis pericarditis, myocarditis / pericarditis, cardiomyopathy, cardiac arrhythmia, cardiac ischemia. The number of patients in the ID group developing Covid-19 related complications while in hospital were compared to controls using Fisher's exact test.

For peer review only

Appendix Figure 1. Mortality rates of hospitalised COVID-19 patients with and without an intellectual disability diagnosis

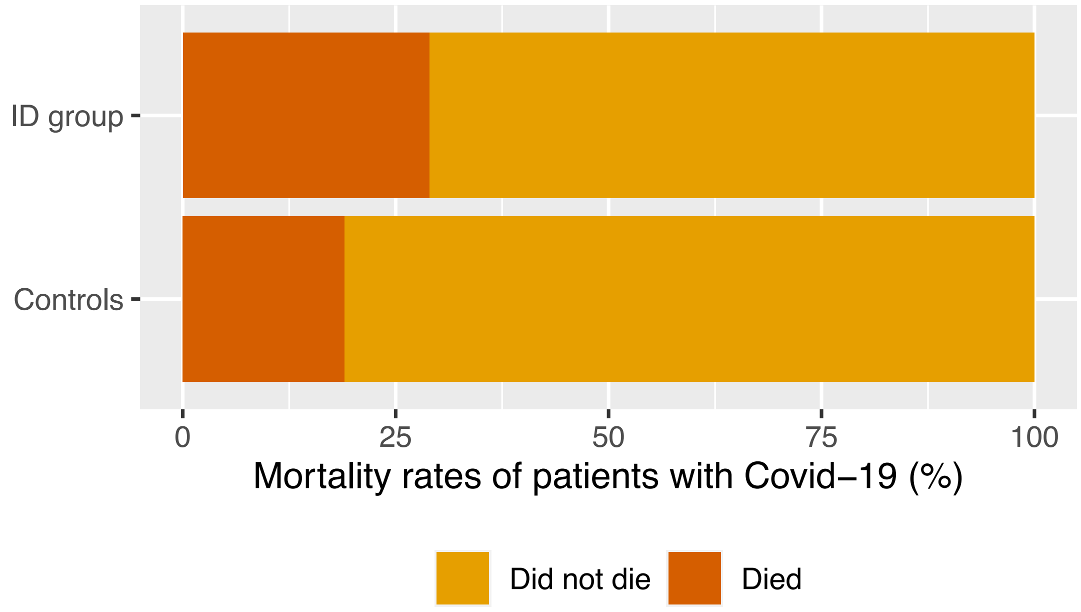

Appendix Table 2. Factors associated with mortality in hospitalised COVID-19 patients

	Risk ratio	95% CI	p value
Age group			
20-29 years old	3.39	0.40 - 44.51	0.300
30-39 years old	6.95	1.29 - 61.92	*0.064*
40-49 years old	10.17	2.06 - 72.36	**0.023**
50-59 years old	22.22	5.44 - 90.88	**0.001**
60-69 years old	25.94	6.57 - 93.93	**0.0006**
70-79 years old	37.26	10.31 - 100.09	**0.0001**
80+ years old	60.18	20.83 - 106.13	**<0.0001**
Male sex	1.18	0.918 - 1.50	0.191
Chronic cardiac disease	1.35	1.02 - 1.76	**0.038**
Chronic pulmonary disease	1.66	1.22 - 2.16	**0.002**
Chronic kidney disease	1.58	1.13 - 2.12	**0.009**
Liver disease	1.69	0.97 - 2.54	*0.055*
Obesity	1.21	0.97 - 1.62	0.242
Chronic neurological disorder	1.64	1.23 - 2.11	**0.001**
Dementia	1.11	0.71 - 1.64	0.632
Malignant neoplasm	1.32	0.84 - 1.92	0.209
Shortness of breath	0.96	0.70 - 1.29	0.785
Respiratory rate	1.03	1.02 - 1.05	**0.0003**
No oxygen therapy	0.72	0.55 - 0.91	**0.005**
Admission to ICU	0.92	0.51 - 1.51	0.758
Intubation	3.11	2.22 - 3.98	**<0.0001**
Non-invasive respiratory support	1.44	1.02 - 1.95	**0.039**
DS diagnosis	1.92	1.19 - 2.76	**0.009**
ID diagnosis	1.56	1.17 - 2.02	**0.003**

ICU, Intensive Care Unit; DS, Down syndrome, ID; Intellectual disability. Tracheostomy was not included in the model due to a large proportion of missing data.

For peer review only

Appendix Table 3. Factors associated with mortality in hospitalised COVID-19 patients with an intellectual disability diagnosis

	**Risk ratio**	**95% CI**	**p value**
Age group			
20-29 years old	1.12	0.13 - 4.99	0.903
30-39 years old	2.41	0.61 - 6.58	0.213
40-49 years old	2.85	0.80 – 7.00	0.120
50-59 years old	4.28	1.62 - 8.04	**0.012**
60-69 years old	6.43	3.21 - 9.07	**0.0002**
70-79 years old	4.04	1.40 - 7.93	**0.022**
80+ years old	7.33	3.74 - 9.44	**0.0001**
Male sex	1.24	0.84 - 1.71	0.267
DS diagnosis	1.41	0.86 - 2.02	0.152
Chronic cardiac disease	1.50	0.94 - 2.12	*0.085*
Chronic pulmonary disease	1.08	0.55 - 1.78	0.789
Chronic kidney disease	1.50	0.85 - 2.22	0.142
Liver disease	1.07	0.33 - 2.20	0.894
Obesity	0.93	0.48 - 1.52	0.803
Chronic neurological disorder	1.39	0.94 - 1.90	*0.091*
Dementia	1.25	0.66 - 2.01	0.454
Malignant neoplasm	0.81	0.28 - 1.69	0.633
Shortness of breath	0.99	0.62 - 1.47	0.960
Respiratory rate	1.02	1.00 - 1.05	**0.036**
No oxygen therapy	0.49	0.31 - 0.73	**0.0002**
Access to any intervention	1.54	0.99 - 2.15	*0.054*

For peer review only

Appendix Table 4. Associations between complications due to COVID-19 and mortality in patients with an ID diagnosis

	Risk ratio	95% CI	*p value*
Age group			
20-29 years old	2.39	0.22 - 22.71	0.478
30-39 years old	4.78	0.79 - 27.72	0.141
40-49 years old	7.00	1.39 - 30.29	*0.053*
50-59 years old	7.92	1.77 - 30.94	**0.032**
60-69 years old	14.97	4.16 -34.30	**0.003**
70-79 years old	7.37	1.52 - 30.59	**0.045**
80+ years old	18.11	5.11 -35.12	**0.001**
Male sex	1.11	0.77 - 1.54	0.550
Viral pneumonia	2.74	1.97 - 3.60	**<0.0001**
Bacterial pneumonia	1.60	0.98 - 2.29	*0.054*
Acute respiratory syndrome	2.07	1.28 - 2.88	**0.006**
Other lung complications[1]	1.76	0.93 -2.	*0.077*
Seizures	0.39	0.06 - 1.16	0.146
Other neurological complications[2]	0.87	0.15 - 2.50	0.844
Other cardiac complications[3]	0.64	0.24 -1.34	0.278
Bacteraemia	1.57	0.42 - 3.02	0.432
Gastrointestinal hemorrhage or coagulation disorder	0.51	0.12 -1.48	0.267
Anaemia	0.51	0.20 - 1.08	*0.096*
Acute renal injury / Acute renal failure	1.17	0.62 - 1.92	0.594
Liver dysfunction	0.90	0.21 - 2.11	0.851
Hypoglycaemia or hyperglycaemia	0.53	0.17 -1.25	0.183

[1]Combined cryptogenic organizing pneumonia (COP), pneumothorax, pleural effusion and bronchiolitis, [2]Combined Stroke / Cerebrovascular accident and other neurological complication, [3]Combined congestive heart failure, endocarditis / myocarditis pericarditis, myocarditis / pericarditis, cardiomyopathy, cardiac arrhythmia, cardiac ischemia. Meningitis, pancreatitis and rhabdomyolysis where removed from the model because they were recorded in less than 1% of ID patients. Ethnicity and cardiac arrest were also removed because they were not good predictors in the model.

For peer review only

Appendix Table 5. Associations between complications due to COVID-19 and mortality in patients without an ID diagnosis

	Risk ratio	95% CI	*p value*
Age group			
20-29 years old	1.07	0.042 - 22.07	0.961
30-39 years old	2.04	0.30 - 29.80	0.522
40-49 years old	4.12	0.75 - 45.82	0.179
50-59 years old	10.34	2.34 - 69.97	**0.018**
60-69 years old	13.12	3.05 - 76.02	**0.008**
70-79 years old	26.96	7.05 -91.13	**0.0005**
80+ years old	36.49	10.18 - 95.93	**0.0001**
Male sex	1.02	0.75 - 1.35	0.913
Viral pneumonia	1.56	1.16 - 2.07	**0.003**
Bacterial pneumonia	1.01	0.63 - 1.52	0.970
Acute respiratory syndrome	1.91	1.32 - 2.62	**0.0008**
Other lung complications[1]	1.11	0.64 - 1.79	0.683
Seizures	0.97	0.32 - 2.23	0.958
Other neurological complications[2]	0.93	0.33 - 2.06	0.881
Cardiac arrest	5.38	3.94 - 6.15	**<0.0001**
Other cardiac complications[3]	1.82	1.22 - 2.57	**0.004**
Bacteraemia	0.82	0.32 - 1.75	0.646
Gastrointestinal hemorrhage or coagulation disorder	2.78	1.60 - 4.09	**0.0009**
Anaemia	1.23	0.80 - 1.80	0.316
Acute renal injury / Acute renal failure	1.99	1.41 - 2.69	**0.0002**
Liver dysfunction	0.50	0.21 - 1.03	*0.072*
Hypoglycaemia or hyperglycaemia	1.15	0.64 - 1.90	0.620

[1]Combined cryptogenic organizing pneumonia (COP), pneumothorax, pleural effusion and bronchiolitis, [2]Combined Stroke / Cerebrovascular accident and other neurological complication, [3]Combined congestive heart failure, endocarditis / myocarditis pericarditis, myocarditis / pericarditis, cardiomyopathy, cardiac arrhythmia, cardiac ischemia. Meningitis, pancreatitis and rhabdomyolysis where removed from the model because they were recorded in less than 1% of control patients. Ethnicity was removed because it was not a good predictor in the model.

For peer review only

Appendix Table 6. Kaplan-Meier estimates of survival probability of hospitalised COVID-19 patients with and without an intellectual disability diagnosis

Time in hospital (days)	Survival probability (% and 95% CI)	
	Controls (n = 1484)	ID group (n = 472)
0	99.6 (99.3 - 99.9)	98.7 (97.7 - 99.7)
5	93.5 (92.1 - 95.0)	83.4 (79.9 - 87.0)
10	83.0 (80.5 - 85.6)	70.9 (66.5 - 75.7)
15	73.6 (70.3 - 77.0)	65.6 (60.7 - 70.8)
20	67.3 (63.5 - 71.3)	60.7 (55.4 - 66.6)

Appendix Figure 2. Violin plot of the distribution of length of stay in COVID-19 patients with and without an intellectual disability diagnosis who were discharged alive

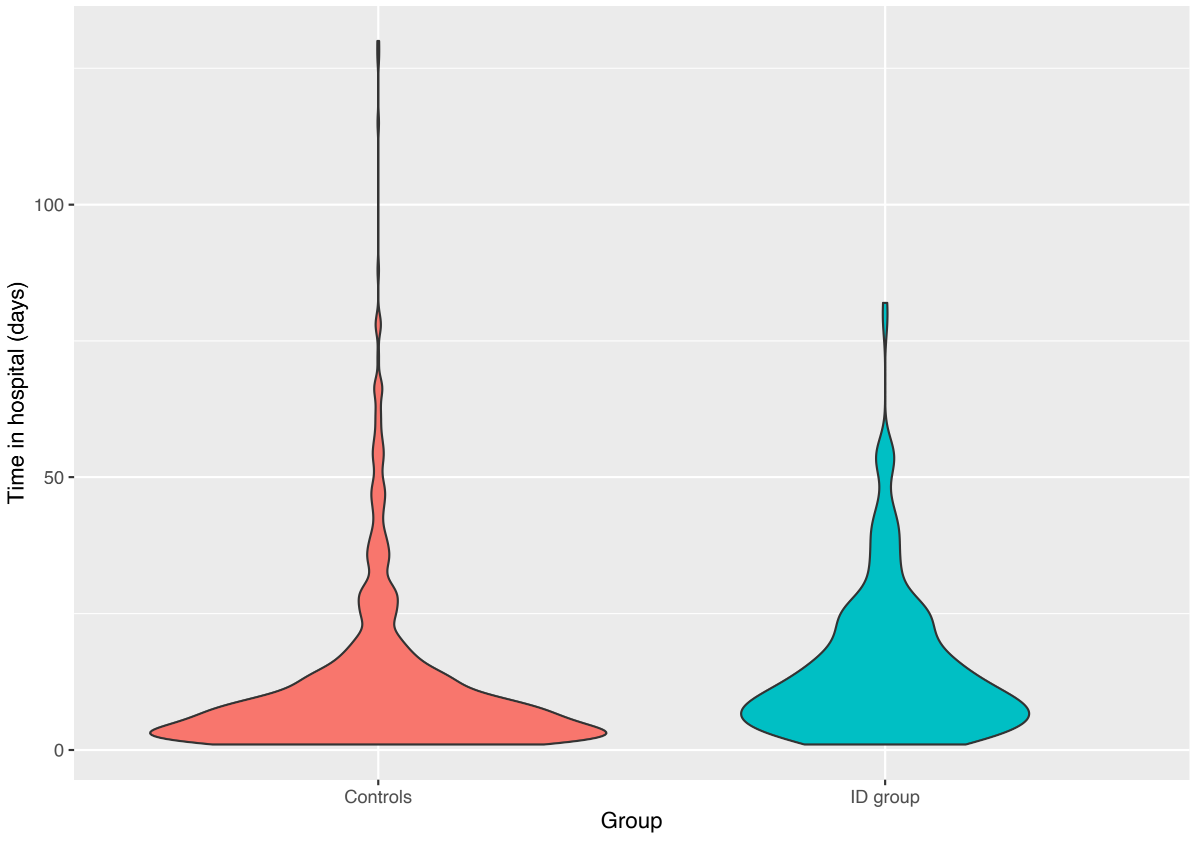

The RECORD statement – checklist of items, extended from the STROBE statement, that should be reported in observational studies using routinely collected health data.

	Item No.	STROBE items	Location in manuscript where items are reported	RECORD items	Location in manuscript where items are reported
**Title and abstract**					
	1	(a) Indicate the study's design with a commonly used term in the title or the abstract (b) Provide in the abstract an informative and balanced summary of what was done and what was found	2	RECORD 1.1: The type of data used should be specified in the title or abstract. When possible, the name of the databases used should be included.<br><br>RECORD 1.2: If applicable, the geographic region and timeframe within which the study took place should be reported in the title or abstract.<br><br>RECORD 1.3: If linkage between databases was conducted for the study, this should be clearly stated in the title or abstract.	2
**Introduction**					
Background rationale	2	Explain the scientific background and rationale for the investigation being reported	4		
Objectives	3	State specific objectives, including any prespecified hypotheses	4		
**Methods**					
Study Design	4	Present key elements of study design early in the paper	5		
Setting	5	Describe the setting, locations, and relevant dates, including periods of recruitment, exposure, follow-up, and data collection	5		

For peer review only

Participants	6	*(a) Cohort study* - Give the eligibility criteria, and the sources and methods of selection of participants. Describe methods of follow-up<br>*Case-control study* - Give the eligibility criteria, and the sources and methods of case ascertainment and control selection. Give the rationale for the choice of cases and controls<br>*Cross-sectional study* - Give the eligibility criteria, and the sources and methods of selection of participants<br><br>*(b) Cohort study* - For matched studies, give matching criteria and number of exposed and unexposed<br>*Case-control study* - For matched studies, give matching criteria and the number of controls per case	5	RECORD 6.1: The methods of study population selection (such as codes or algorithms used to identify subjects) should be listed in detail. If this is not possible, an explanation should be provided.<br><br>RECORD 6.2: Any validation studies of the codes or algorithms used to select the population should be referenced. If validation was conducted for this study and not published elsewhere, detailed methods and results should be provided.<br><br>RECORD 6.3: If the study involved linkage of databases, consider use of a flow diagram or other graphical display to demonstrate the data linkage process, including the number of individuals with linked data at each stage.	5
Variables	7	Clearly define all outcomes, exposures, predictors, potential confounders, and effect modifiers. Give diagnostic criteria, if applicable.	5	RECORD 7.1: A complete list of codes and algorithms used to classify exposures, outcomes, confounders, and effect modifiers should be provided. If these cannot be reported, an explanation should be provided.	n/a
Data sources/ measurement	8	For each variable of interest, give sources of data and details of methods of assessment (measurement).<br>Describe comparability of assessment methods if there is more than one group	5		

For peer review only

Bias	9	Describe any efforts to address potential sources of bias	5/6		
Study size	10	Explain how the study size was arrived at	5		
Quantitative variables	11	Explain how quantitative variables were handled in the analyses. If applicable, describe which groupings were chosen, and why	n/a		
Statistical methods	12	(a) Describe all statistical methods, including those used to control for confounding<br>(b) Describe any methods used to examine subgroups and interactions<br>(c) Explain how missing data were addressed<br>(d) *Cohort study* - If applicable, explain how loss to follow-up was addressed<br>*Case-control study* - If applicable, explain how matching of cases and controls was addressed<br>*Cross-sectional study* - If applicable, describe analytical methods taking account of sampling strategy<br>(e) Describe any sensitivity analyses	6		
Data access and cleaning methods		..		RECORD 12.1: Authors should describe the extent to which the investigators had access to the database population used to create the study population.	6

For peer review only

				RECORD 12.2: Authors should provide information on the data cleaning methods used in the study.	
Linkage		..		RECORD 12.3: State whether the study included person-level, institutional-level, or other data linkage across two or more databases. The methods of linkage and methods of linkage quality evaluation should be provided.	
**Results**					
Participants	13	(a) Report the numbers of individuals at each stage of the study (*e.g.*, numbers potentially eligible, examined for eligibility, confirmed eligible, included in the study, completing follow-up, and analysed)<br>(b) Give reasons for non-participation at each stage.<br>(c) Consider use of a flow diagram	6	RECORD 13.1: Describe in detail the selection of the persons included in the study (*i.e.,* study population selection) including filtering based on data quality, data availability and linkage. The selection of included persons can be described in the text and/or by means of the study flow diagram.	n/a
Descriptive data	14	(a) Give characteristics of study participants (*e.g.*, demographic, clinical, social) and information on exposures and potential confounders<br>(b) Indicate the number of participants with missing data for each variable of interest<br>(c) *Cohort study* - summarise follow-up time (*e.g.*, average and total amount)	6/7		
Outcome data	15	*Cohort study* - Report numbers of outcome events or summary measures over time<br>*Case-control study* - Report numbers in each exposure	6/7		

For peer review only

		category, or summary measures of exposure<br>*Cross-sectional study* - Report numbers of outcome events or summary measures			
Main results	16	(a) Give unadjusted estimates and, if applicable, confounder-adjusted estimates and their precision (e.g., 95% confidence interval). Make clear which confounders were adjusted for and why they were included<br>(b) Report category boundaries when continuous variables were categorized<br>(c) If relevant, consider translating estimates of relative risk into absolute risk for a meaningful time period	10-13		
Other analyses	17	Report other analyses done—e.g., analyses of subgroups and interactions, and sensitivity analyses	n/a		
**Discussion**					
Key results	18	Summarise key results with reference to study objectives	13		
Limitations	19	Discuss limitations of the study, taking into account sources of potential bias or imprecision. Discuss both direction and magnitude of any potential bias	14	RECORD 19.1: Discuss the implications of using data that were not created or collected to answer the specific research question(s). Include discussion of misclassification bias, unmeasured confounding, missing data, and changing eligibility over time, as they pertain to the study being reported.	14
Interpretation	20	Give a cautious overall interpretation of results considering objectives,	15		

For peer review only

		limitations, multiplicity of analyses, results from similar studies, and other relevant evidence			
Generalisability	21	Discuss the generalisability (external validity) of the study results	15		
**Other Information**					
Funding	22	Give the source of funding and the role of the funders for the present study and, if applicable, for the original study on which the present article is based	16		
Accessibility of protocol, raw data, and programming code		..	16	RECORD 22.1: Authors should provide information on how to access any supplemental information such as the study protocol, raw data, or programming code.	16

*Reference: Benchimol EI, Smeeth L, Guttmann A, Harron K, Moher D, Petersen I, Sørensen HT, von Elm E, Langan SM, the RECORD Working Committee. The REporting of studies Conducted using Observational Routinely-collected health Data (RECORD) Statement. *PLoS Medicine* 2015; in press.

*Checklist is protected under Creative Commons Attribution (CC BY) license.

For peer review only
